# Virtual-Array Implementation for High Performance Multi-Target Angular Sensing in UAV-ISAC Systems

**DOI:** 10.3390/s26196221

**Published:** 2026-09-30

**Authors:** Jing Zhang, Yongwei Zhang, Zhaozong Meng, Orhan Kaplan

**Affiliations:** 1 School of Transportation and Civil Engineering, Nantong University, Nantong 226019, China; 2 Department of Mechanical Engineering, Hebei University of Technology, Tianjin 300401, China; 3 Electrical and Electronic Engineering, Faculty of Technology, Gazi University, 06560 Ankara, Turkey

**Keywords:** angular resolution, angle estimation, ISAC, MIMO-OFDM, UAV, virtual array

## Abstract

UAV-enabled Integrated Sensing and Communication (ISAC) systems simultaneously support target sensing and communication and, combined with their abilities in autonomy and control situations, they have the potential to be deployed for search and rescue tasks that were, previously, challenging or impossible. However, the limited apertures of UAV-mounted physical arrays restrict angular resolution and multi-target sensing capability. This work proposes a method for implementing virtual arrays in UAV-assisted MIMO-OFDM ISAC systems to enhance the multi-target sensing capability. With a range of possibilities including transmit–receive channel pairings and two virtual-array configurations, Virtual-7 and Virtual-10 are developed to form greater effective apertures out of a 4×4 ISAC transceiver. A sensing model based on virtual arrays and the corresponding signal-processing procedure are established for angle estimation and for sensing-performance investigation. Simulation results show that ISAC systems with virtual arrays perform better in terms of multi-target detection. The proposed method for virtual-array establishment can, potentially, be applied in UAV-ISACs for high-resolution sensing with a low-profile configuration requirement.

## 1. Introduction

Integrated sensing and communication (ISAC) integrates wireless communication and environmental sensing within a unified framework. Compared with conventional separate communication and radar systems, ISACs can improve spectrum utilization, reduce hardware complexity, and enhance environmental sensing capability by sharing spectrum, hardware, and signal-processing modules [1,2]. Therefore, ISACs have broad application prospects in intelligent transportation, target monitoring, emergency communication, disaster relief, space–air–ground integrated networks, vehicular networks, and millimeter-wave radar-communication systems [3,4].

Unmanned aerial vehicles (UAVs), with flexible deployment, high mobility, wide coverage, and favorable air-to-ground line-of-sight propagation, are promising platforms for ISACs [5]. A UAV can act as an aerial ISAC base station, serving ground-communication users while sensing targets and estimating their parameters. Existing studies on mobile-user tracking, robust beamforming, and trajectory optimization have demonstrated the deployment flexibility of UAV-ISAC systems [6,7]. Multi-UAV cooperative placement and power control further show that spatial resources can be flexibly configured according to mission requirements [8].

However, constrained by size, weight, power consumption, and payload capacity, UAVs are difficult to equip with large-scale antenna arrays. Although small-scale physical arrays are easier to implement, their limited aperture and spatial degrees of freedom may degrade direction-of-arrival (DoA) estimation accuracy and multi-target angular resolution, especially when multiple targets are closely spaced in the angular domain. Existing studies have shown that array topology directly affects the angular-sensing performance of ISAC systems [9], while aperture length and antenna placement are also important factors that determine sensing resolution [10]. Therefore, how to improve the equivalent aperture and angular-resolution capability without significantly increasing the hardware burden of UAV platforms is a key problem.

Virtual arrays provide an effective approach to overcoming the aperture limitation of physical arrays. In a multiple-input multiple-output (MIMO) architecture, an equivalent virtual array can be formed by exploiting the combinations between transmit and receive antenna elements. Early MIMO radar studies have shown that multiple transmit and receive antennas can increase system degrees of freedom and improve spatial resolution [11]. In colocated MIMO radar, virtual arrays can expand the equivalent array aperture without significantly increasing the number of physical antennas, thereby improving DoA-estimation accuracy and multi-target separation capability [12], and they are particularly suitable for UAV-ISAC systems constrained by antenna number and platform payload.

In recent years, ISAC architectures, waveform design, and multi-antenna sensing have been widely studied. Waveform design in dual-functional radar-communication systems is an important technique for integrating communication and sensing [13]. MIMO-orthogonal frequency division multiplexing (MIMO-OFDM) ISAC systems can use OFDM waveforms for both communication and target range, Doppler, and angle estimation [14,15]. Optimized precoding and uplink MIMO-OFDM radar-communication architectures have further demonstrated the effectiveness of OFDM for multi-antenna joint communication and sensing [16].

In OFDM-ISAC studies, code-division OFDM and in-band full-duplex OFDM-ISAC schemes have further promoted the development of waveform multiplexing and multi-dimensional parameter estimation techniques [17,18]. Recent tutorials and surveys on MIMO-OFDM ISAC systems have also systematically summarized its research progress in terms of communication transmission, target-range estimation, Doppler estimation, and angle estimation [19]. In addition, for high-mobility scenarios, orthogonal time frequency space (OTFS) modulation has been introduced into ISAC systems for delay–Doppler domain signal processing and target-parameter estimation, providing new insights for ISAC system design in dynamic environments [20]. These studies indicate that multi-transmit and multi-receive antenna structures can provide richer spatial observations, which also lays the foundation for virtual-array construction in ISAC systems.

With the development of ISACs, virtual arrays have gradually been introduced into dual-functional radar-communication and ISAC systems. OTFS-based dual-functional MIMO radar-communication systems have shown that higher sensing resolution than that of physical receive arrays can be achieved by exploiting the equivalent virtual aperture formed by MIMO structures [21]. Related studies have further constructed virtual arrays through specific time-frequency or delay–Doppler resource designs while maintaining communication transmission, thereby improving target-parameter-estimation performance [22]. In monostatic MIMO-ISAC scenarios, quasi-orthogonal space-frequency block coding (SFBC) and SFBC-based virtual-array schemes can enhance the separability of signals transmitted from different antennas, thus supporting virtual-array construction [23,24]. These studies demonstrate that virtual arrays can serve as an important approach for improving ISACs’ sensing resolution.

Besides waveform and coding methods, array structure is also an important factor affecting ISACs’ sensing performance. Hybrid physical- and virtual-array-based OFDM-ISAC studies have shown that combining physical and virtual arrays can improve sensing capability in complex scenarios [25]. Joint DOA and time-of-arrival (TOA) estimation further reflects the relationship between array structures and multi-dimensional target-parameter estimation [26]. DOA-estimation-oriented array partitioning and beamforming designs indicate that array-resource allocation and beam control directly affect angle-estimation performance [27].

With limited hardware resources, sparse MIMO arrays and antenna selection also provide useful approaches for ISAC-array design. Sparse MIMO arrays can improve sensing performance by enlarging the equivalent aperture and spatial degrees of freedom [28]. Deep-learning-based antenna selection and hybrid beamforming can further improve the utilization of limited antenna resources [29]. In addition, multiple-target detection in cell-free massive MIMO-assisted ISACs show that cooperative multi-antenna structures can further enhance multi-target sensing capability in complex networked scenarios [30].

Although the above studies provide important foundations for ISAC-array design and virtual-array applications, several issues still remain. First, most existing works focus on virtual-array construction, sparse-array optimization, antenna selection, beamforming design, or overall sensing-performance improvement, while the angular-resolution capability of regular MIMO virtual arrays in resource-constrained UAV-ISAC platforms has not been sufficiently investigated. Second, although sparse-array methods can effectively expand the aperture, they usually require further consideration of array-geometry optimization, grating-lobe control, side-lobe suppression, and sparse-reconstruction complexity. In contrast, regular MIMO virtual arrays are easier to model and implement on small-scale UAV platforms, and they are more suitable for directly analyzing the influence of different transmit–receive antenna-placement strategies on angular resolution. Especially when two targets are closely spaced in the angular domain, the effects of different numbers of virtual elements, equivalent apertures, and element distributions on two-target resolvability still lack systematic comparison.

To address these issues, this paper investigates the improvement in sensing in terms of angular resolution using MIMO virtual arrays in a resource-constrained UAV-ISAC scenario. An OFDM-based UAV-ISAC simulation platform is constructed, where a hovering UAV acts as an aerial ISAC base station and simultaneously serves a ground-communication user and multiple sensing targets. In the sensing-processing stage, multi-transmit and multi-receive antenna channels are used to construct virtual-array data for DOA estimation and two-target angular-resolution analysis. The virtual array in this paper is mainly applied to sensing-domain received-data processing to expand the equivalent sensing aperture and improve angular resolution without changing the basic structure of the communication link. This paper does not aim at sparse-array optimization or complex sparse reconstruction. Instead, it focuses on the feasibility and angular-resolution performance differences of small-scale regular MIMO virtual arrays in UAV-ISAC scenarios.

The main contributions of this paper are summarized as follows.

1. To address the angular-resolution limitation caused by the restricted number of physical antennas on UAV platforms, MIMO virtual arrays are used to expand the equivalent sensing aperture without significantly increasing the physical antenna scale, thereby improving DOA-estimation accuracy and multi-target angular resolution.
2. In contrast to sparse-array optimization approaches, this paper focuses on regular MIMO arrays suitable for small-scale UAV platforms. Different transmit–receive-element arrangements are used to construct (7×1) and (10×1) virtual arrays, which are compared with the (4×1) physical array to analyze the effects of virtual-element number, equivalent aperture, and element distribution on angular resolution.
3. By varying the angular separation between two targets, the DOA-estimation and target-resolution capabilities of different array structures are systematically compared.
4. Multiple performance metrics, including DOA root mean square error (RMSE), root Cramér–Rao bound (root CRB), probability of resolution ($P_{r}$), and conditional DOA RMSE (conditional RMSE), are used to evaluate the angle-estimation accuracy, theoretical performance bound, and two-target resolution capability of different array structures.

The results show that, compared with the (4×1) physical array, virtual arrays improve angle-estimation accuracy and target resolution in UAV-ISAC systems. Among the three array configurations, the (10×1) virtual array achieves the best sensing performance due to its larger effective aperture and greater number of distinct virtual elements. These results verify the effectiveness of virtual arrays for improving angular resolution in resource-constrained UAV-ISAC systems.

## 2. ISAC Sensor-System Model

### 2.1. System Architecture

**Figure 1:**
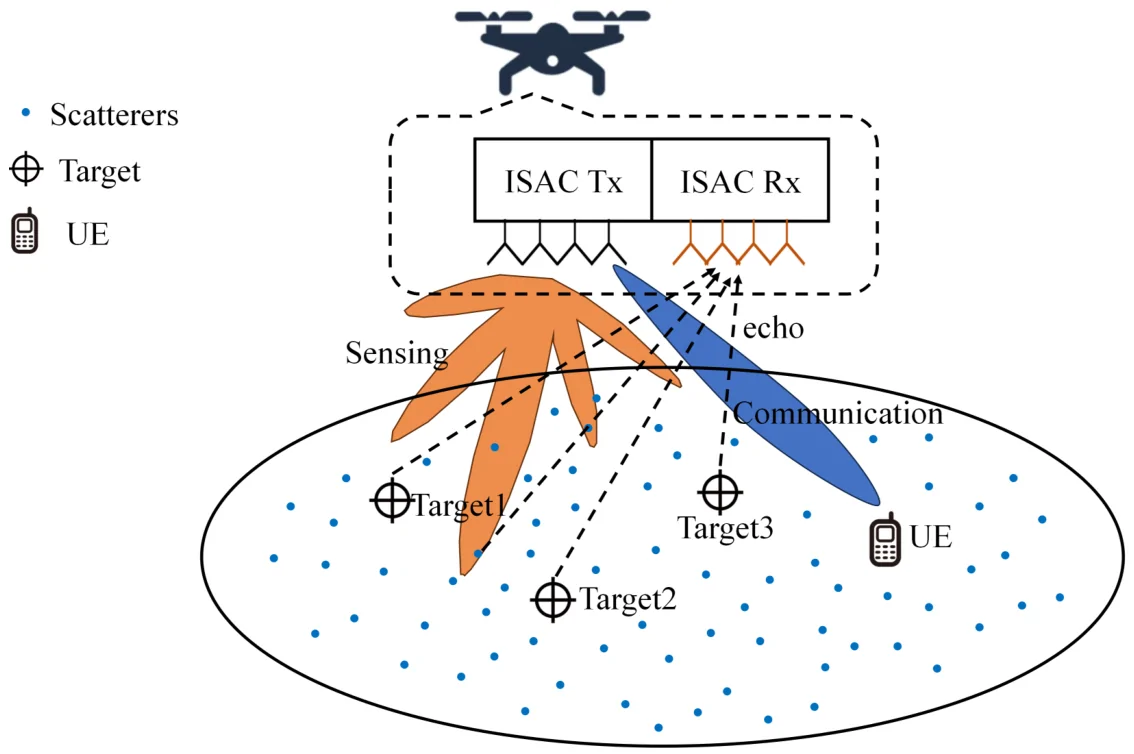
Architecture of a communication-centric downlink ISAC system with colocated Tx/Rx arrays enabling monostatic sensing.

We consider a communication-centric downlink ISAC system, as illustrated in Figure 1. The system consists of a hovering UAV carrying a multi-antenna base station (BS), a user equipment (UE), and multiple sensing targets and scatterers. The UAV-BS is equipped with $N_{t}$ transmit antennas and $N_{r}$ receive antennas to support communication and sensing.

In the considered system, the UAV-BS transmits downlink signals to the UE via its multi-antenna transmit array to enable wireless communication. Meanwhile, the UAV-BS exploits a colocated receive array to collect echoes reflected from the environmental targets and scatterers, thereby realizing monostatic radar sensing. The sensing functionality aims to detect targets in the environment and to estimate their key parameters, including range, velocity, and angle.

### 2.2. Signal Model

#### 2.2.1. Transmit-Signal Model

The considered system for study adopts a MIMO–OFDM integrated sensing and communication (ISAC) transmission framework. The transmitter is equipped with $N_{t}$ transmit antennas and employs $N_{c}$ OFDM subcarriers. Within a coherent processing interval (CPI) of duration $N_{s}T_{\mathrm{sym}}$, where $N_{s}$ denotes the number of OFDM symbols and $T_{\mathrm{sym}}$ denotes the OFDM-symbol duration, the relevant channel parameters are assumed to remain approximately constant.

Let ${\tilde{x}}_{n,m}\in C^{N_{t}\times 1}$ denote the ISAC transmit-signal vector on subcarrier $n=0,\ldots ,N_{c}-1$ and OFDM-symbol index $m=0,\ldots ,N_{s}-1$. Let *P* denote the total transmit-power budget, and let α∈[0,1] denote the power-allocation factor between communication and sensing. The transmit signal is expressed as

(1) $${\tilde{x}}_{n,m}=\sqrt{\alpha P}\;w_{c}s_{n,m}^{c}+\sqrt{(1-\alpha )P}\;x_{n,m}^{s},$$

where $w_{c}\in C^{N_{t}\times 1}$ denotes the communication beamforming vector, $s_{n,m}^{c}$ denotes the communication data or pilot symbol, and $x_{n,m}^{s}\in C^{N_{t}\times 1}$ denotes the normalized sensing transmit vector. The communication symbol and beamforming vector satisfy

(2) $$E\;\left[|s_{n,m}^{c}{|}^{2}\right]=1,\;{∥w_{c}∥}_{2}^{2}=1.$$

Accordingly, αP and (1−α)P represent the power budgets allocated to the communication and sensing components, respectively. The specific design of $w_{c}$ is introduced in the subsequent communication model.

To obtain the transmit-dependent sensing-channel observations required for virtual-array construction, a transmit-orthogonal sensing-acquisition block is embedded in the ISAC frame. Let $s_{i}={\left[s_{i}\left[1\right],\;s_{i}\left[2\right],\;\ldots ,\;s_{i}\left[L_{o}\right]\right]}^{T}\in C^{L_{o}\times 1}$ denote the orthogonal pilot sequence assigned to the *i*th transmit antenna, where $i=1,\ldots ,N_{t}$ and $L_{o}$ denotes the pilot-sequence length. The pilot sequences satisfy

(3) $$s_{i}^{H}s_{j}=\left\{\begin{array}{cc} L_{o}, & i=j,\\ 0, & i\neq j. \end{array}\right.$$

Collecting the pilot sequences of all transmit antennas gives

(4) $$S=\left[\begin{array}{c} s_{1}^{T}\\ s_{2}^{T}\\ \vdots \\ s_{N_{t}}^{T} \end{array}\right]\in C^{N_{t}\times L_{o}},$$

which satisfies $SS^{H}=L_{o}I_{N_{t}}$.

Hence, the pilot length should satisfy $L_{o}\geq N_{t}$ to distinguish the sensing signals associated with the $N_{t}$ transmit antennas. For the $N_{t}=4$ transmit antennas considered in this work, $L_{o}=4$ is adopted, with the Walsh–Hadamard pilot matrix given by

(5) $$S=\left[\begin{array}{cccc} 1 & 1 & 1 & 1\\ 1 & -1 & 1 & -1\\ 1 & 1 & -1 & -1\\ 1 & -1 & -1 & 1 \end{array}\right],$$

which satisfies $SS^{H}=4I_{4}$.

Let $a_{n,m}$ denote the normalized sensing-reference symbol, satisfying $E[|a_{n,m}{|}^{2}]=1$.

Collecting the $L_{o}$ orthogonal pilot dimensions into one sensing-acquisition block gives

(6) $$X_{p}\left[n,m\right]=\sqrt{\frac{(1-\alpha )P}{N_{t}L_{o}}}\;a_{n,m}S,$$

where $X_{p}\left[n,m\right]\in C^{N_{t}\times L_{o}}$.

Since $SS^{H}=L_{o}I_{N_{t}}$, we have ${∥S∥}_{F}^{2}=N_{t}L_{o}$, and, therefore,

(7) $$E\left[{∥X_{p}\left[n,m\right]∥}_{F}^{2}\right]=\left(1-\alpha \right)P.$$

Thus, the orthogonal pilot dimensions provide separability among the transmit-dependent sensing channels while keeping the total sensing energy of the acquisition block fixed at (1−α)P. The corresponding resource cost is the use of $L_{o}$ orthogonal pilot dimensions for transmit-channel separation, while no additional physical transmit or receive antennas or RF chains are required. The sensing channel is assumed to remain approximately unchanged over the $L_{o}$ pilot dimensions, which enables the transmit-dependent sensing channels to be separated using the orthogonality of S.

#### 2.2.2. Communication Model

Consider the frequency-domain MIMO–OFDM communication link, where the communication user is equipped with $N_{u}$ receive antennas. The communication channel on the *n*th subcarrier and the *m*th OFDM symbol is denoted by ${\tilde{H}}_{n,m}^{c}\in C^{N_{u}\times N_{t}}$ and modeled as

(8) $${\tilde{H}}_{n,m}^{c}=\sum_{\ell =1}^{L_{\mathrm{ch}}}b_{\ell }e^{-j2\pi n\tau_{\ell }\Delta f}e^{j2\pi f_{D,\ell }mT_{\mathrm{sym}}}a_{\mathrm{rx}}\left(\phi_{\ell }\right)a_{\mathrm{tx}}^{T}\left(\theta_{\ell }\right),$$

where $L_{\mathrm{ch}}$ denotes the number of dominant propagation paths, $b_{\ell }$ is the complex channel gain of the *ℓ*th path, $\tau_{\ell }$ and $f_{D,\ell }$ denote its propagation delay and Doppler frequency, respectively, $\theta_{\ell }$ and $\phi_{\ell }$ denote the angle of departure (AoD) and angle of arrival (AoA), respectively, and Δf is the subcarrier spacing.

For the considered UAV-to-ground air-to-ground (A2G) communication link, the complex gain of the *ℓ*th path is modeled as

(9) $$b_{\ell }=\sqrt{\frac{h_{0}}{d_{\ell }^{2}}}\left(\sqrt{\frac{\kappa }{\kappa +1}}+\sqrt{\frac{1}{\kappa +1}}{\tilde{g}}_{\ell }\right),$$

where $h_{0}$ denotes the channel gain at the reference distance of 1m, $d_{\ell }$ is the propagation distance of the *ℓ*th path, κ is the Rician factor, and ${\tilde{g}}_{\ell }∼\mathrm{CN}\left(0,1\right)$ denotes the small-scale fading component.

Assuming uniform linear arrays (ULAs) at the transmitter and the communication receiver, the corresponding steering vectors are given by

(10) $$a_{\mathrm{tx}}\left(\theta_{\ell }\right)={\left[1,\;e^{-j2\pi \frac{d_{\mathrm{tx}}}{\lambda }\sin\left(\theta_{\ell }\right)},\;\ldots ,\;e^{-j2\pi \left(N_{t}-1\right)\frac{d_{\mathrm{tx}}}{\lambda }\sin\left(\theta_{\ell }\right)}\right]}^{T},$$

and

(11) $$a_{\mathrm{rx}}\left(\phi_{\ell }\right)={\left[1,\;e^{-j2\pi \frac{d_{\mathrm{rx}}}{\lambda }\sin\left(\phi_{\ell }\right)},\;\ldots ,\;e^{-j2\pi \left(N_{u}-1\right)\frac{d_{\mathrm{rx}}}{\lambda }\sin\left(\phi_{\ell }\right)}\right]}^{T},$$

where $d_{\mathrm{tx}}$ and $d_{\mathrm{rx}}$ denote the inter-element spacings of the transmit and communication receive arrays, respectively, and λ denotes the carrier wavelength.

After synchronization, cyclic-prefix removal, and FFT processing, the received communication signal is

(12) $${\tilde{y}}_{n,m}^{c}={\tilde{H}}_{n,m}^{c}{\tilde{x}}_{n,m}+{\tilde{z}}_{n,m}^{c},$$

where ${\tilde{z}}_{n,m}^{c}∼\mathrm{CN}\left(0,\sigma^{2}I_{N_{u}}\right)$ is the additive white Gaussian noise, with $\sigma^{2}$ denoting the noise power at each receive antenna.

Using the ISAC transmit signal defined in the transmit-signal model, the received signal can be further written as

(13) $${\tilde{y}}_{n,m}^{c}=\sqrt{\alpha P}\;{\tilde{H}}_{n,m}^{c}w_{c}s_{n,m}^{c}+\sqrt{(1-\alpha )P}\;{\tilde{H}}_{n,m}^{c}x_{n,m}^{s}+{\tilde{z}}_{n,m}^{c},$$

where the first term represents the desired communication signal and the second term represents the interference induced by the sensing component.

Let $u_{c}\in C^{N_{u}\times 1}$ denote the receive combining vector. The singular value decomposition (SVD) of the communication channel is given by ${\tilde{H}}_{n,m}^{c}=U\Sigma V^{H}$, where U and V contain the left and right singular vectors, respectively, and Σ contains the singular values.

The communication receive combiner and transmit beamformer are selected as $u_{c}=u_{1}$ and $w_{c}=v_{1}$, respectively, where $u_{1}$ and $v_{1}$ are the left and right singular vectors corresponding to the largest singular value.

The covariance matrix of the normalized sensing-signal vector is defined as

(14) $$R_{s}=E\left[x_{n,m}^{s}{\left(x_{n,m}^{s}\right)}^{H}\right],\;\mathrm{tr}\left(R_{s}\right)=1.$$

Accordingly, the communication SINR on the *n*th subcarrier and the *m*th OFDM symbol is given by

(15) $${\mathrm{SINR}}_{n,m}=\frac{\alpha P{\left|u_{c}^{H}{\tilde{H}}_{n,m}^{c}w_{c}\right|}^{2}}{\left(1-\alpha \right)Pu_{c}^{H}{\tilde{H}}_{n,m}^{c}R_{s}{\left({\tilde{H}}_{n,m}^{c}\right)}^{H}u_{c}+\sigma^{2}}.$$

The corresponding achievable communication rate is

(16) $$R_{n,m}=\log_{2}\left(1+{\mathrm{SINR}}_{n,m}\right),$$

and the average communication rate over $N_{c}$ subcarriers and $N_{s}$ OFDM symbols is

(17) $$\bar{R}=\frac{1}{N_{c}N_{s}}\sum_{n=0}^{N_{c}-1}\sum_{m=0}^{N_{s}-1}R_{n,m}.$$

#### 2.2.3. Sensing Model

Assume that there are *K* sensing targets in the scene. Let $\alpha_{k}$ denote the complex scattering coefficient of the *k*th target, and let $\tau_{k}$ and $f_{D,k}$ denote its round-trip propagation delay and Doppler frequency shift, respectively.

First, from the perspective of an individual transmit–receive antenna pair, the sensing channel between the *i*th transmit antenna and the *p*th receive antenna via the *k*th target can be modeled as

(18) $$g_{k,i,p}\left[n,m\right]=\alpha_{k}e^{-j2\pi n\Delta f\tau_{k}}e^{j2\pi f_{D,k}mT_{\mathrm{sym}}}e^{-j\frac{2\pi }{\lambda }\left(i-1\right)d_{\mathrm{tx}}\sin\theta_{k}}e^{-j\frac{2\pi }{\lambda }\left(p-1\right)d_{\mathrm{rx}}\sin\phi_{k}},$$

where $i=1,\ldots ,N_{t}$ and $p=1,\ldots ,N_{r}$ are the transmit- and sensing-receive-antenna indices, respectively, and $\theta_{k}$ and $\phi_{k}$ denote the AoD and AoA of the *k*th target.

The sensing channel associated with the *k*th target on the *n*th subcarrier and during the *m*th OFDM symbol can be further written in matrix form as

(19) $$G_{k}\left[n,m\right]=\alpha_{k}e^{-j2\pi n\Delta f\tau_{k}}e^{j2\pi f_{D,k}mT_{\mathrm{sym}}}a_{\mathrm{rx}}\left(\phi_{k}\right)a_{\mathrm{tx}}^{T}\left(\theta_{k}\right),$$

where $G_{k}\left[n,m\right]\in C^{N_{r}\times N_{t}}$, $a_{\mathrm{tx}}\left(\theta_{k}\right)\in C^{N_{t}\times 1}$, and $a_{\mathrm{rx}}\left(\phi_{k}\right)\in C^{N_{r}\times 1}$ denote the transmit and receive steering vectors, respectively. Their mathematical forms follow the same uniform linear-array definition as in the communication model, except that the dimension of the receive steering vector is $N_{r}$ rather than $N_{u}$.

Under the co-located far-field assumption, the departure angle and arrival angle can be approximately regarded as identical, i.e.,

(20) $$\theta_{k}\approx \phi_{k}\approx \vartheta_{k}.$$

Accordingly, the sensing channel in (19) can be simplified as

(21) $$G_{k}\left[n,m\right]=\alpha_{k}e^{-j2\pi n\Delta f\tau_{k}}e^{j2\pi f_{D,k}mT_{\mathrm{sym}}}a_{\mathrm{rx}}\left(\vartheta_{k}\right)a_{\mathrm{tx}}^{T}\left(\vartheta_{k}\right).$$

When multiple targets are present, the overall sensing channel is given by the superposition of the contributions from all targets, namely,

(22) $$G\left[n,m\right]=\sum_{k=1}^{K}G_{k}\left[n,m\right].$$

Similar to the target echo channel model, the effective echo channel corresponding to the *ℓ*th clutter or non-target scatterer can be written as

(23) $$c_{\ell ,i,p}\left[n,m\right]=\beta_{\ell }e^{-j2\pi n\Delta f\tau_{\ell }}e^{j2\pi f_{D,\ell }mT_{\mathrm{sym}}}e^{-j\frac{2\pi }{\lambda }\left(i-1\right)d_{\mathrm{tx}}\sin\theta_{\ell }}e^{-j\frac{2\pi }{\lambda }\left(p-1\right)d_{\mathrm{rx}}\sin\phi_{\ell }},$$

where $\beta_{\ell }$, $\tau_{\ell }$, $f_{D,\ell }$, $\theta_{\ell }$, and $\phi_{\ell }$ denote the complex scattering coefficient, delay, Doppler shift, AoD, and AoA of the *ℓ*th component, respectively. The ground and environmental scattering region is modeled by *L* spatially distributed clutter or non-target components. For a hovering or quasi-static UAV, stationary ground clutter satisfies $f_{D,\ell }\approx 0$, while $f_{D,\ell }$ is retained to accommodate possible moving non-target scatterers. Accordingly, the aggregate clutter and non-target scattering-channel matrix is expressed as

(24) $$C\left[n,m\right]=\sum_{\ell =1}^{L}\beta_{\ell }e^{-j2\pi n\Delta f\tau_{\ell }}e^{j2\pi f_{D,\ell }mT_{\mathrm{sym}}}a_{\mathrm{rx}}\left(\phi_{\ell }\right)a_{\mathrm{tx}}^{T}\left(\theta_{\ell }\right).$$

According to the transmit-orthogonal sensing-acquisition signal defined in the transmit-signal model, the received signal at the *p*th sensing receive antenna on the *n*th subcarrier and during the *m*th OFDM symbol, corresponding to the *l*th orthogonal pilot dimension, can be expressed as

(25) $$\begin{array}{cc} y_{p}\left[n,m,l\right] & =\sqrt{\frac{(1-\alpha )P}{N_{t}L_{o}}}a_{n,m}\sum_{i=1}^{N_{t}}\left(\sum_{k=1}^{K}g_{k,i,p}\left[n,m\right]+\sum_{\ell =1}^{L}c_{\ell ,i,p}\left[n,m\right]\right)\\ \; & \;\times s_{i}\left[l\right]+w_{p}\left[n,m,l\right], \end{array}$$

where $l=1,\ldots ,L_{o}$, $s_{i}\left[l\right]$ is the *l*th pilot symbol assigned to transmit antenna *i*, and $w_{p}\left[n,m,l\right]$ is the noise sample. The known reference symbol $a_{n,m}$ can be compensated during channel recovery, while the normalization ensures a total sensing energy of (1−α)P.

By stacking the received samples $y_{p}\left[n,m,l\right]$ over all $p=1,\ldots ,N_{r}$ receive antennas and $l=1,\ldots ,L_{o}$ orthogonal pilot dimensions, the received sensing data matrix is defined as

(26) $$Y\left[n,m\right]=\left(G[n,m]+C[n,m]\right)X_{p}\left[n,m\right]+N\left[n,m\right].$$

Using the pilot orthogonality $SS^{H}=L_{o}I_{N_{t}}$, matched filtering gives

(27) $$\frac{1}{L_{o}}Y\left[n,m\right]S^{H}=\sqrt{\frac{(1-\alpha )P}{N_{t}L_{o}}}a_{n,m}\left(G[n,m]+C[n,m]\right)+\tilde{N}\left[n,m\right],$$

where $\tilde{N}\left[n,m\right]=N\left[n,m\right]S^{H}/L_{o}$ denotes the noise term after matched filtering.

Compensating the known reference symbol $a_{n,m}$ yields the separated transmit–receive channel observation

(28) $$\hat{G}\left[n,m\right]=\frac{\sqrt{N_{t}}}{a_{n,m}\sqrt{L_{o}\left(1-\alpha \right)P}}Y\left[n,m\right]S^{H}.$$

The matrix $\hat{G}\left[n,m\right]\in C^{N_{r}\times N_{t}}$ contains the recovered observations of all $N_{t}N_{r}$ transmit–receive antenna pairs and can be written as

(29) $$\hat{G}\left[n,m\right]=G\left[n,m\right]+C\left[n,m\right]+\bar{N}\left[n,m\right],$$

where $\bar{N}\left[n,m\right]$ denotes the equivalent noise.

Assuming independent entries of variance $\sigma_{g}^{2}$, the equivalent noise power is

(30) $$E\;\left[{∥\bar{N}\left[n,m\right]∥}_{F}^{2}\right]=N_{r}N_{t}\sigma_{g}^{2}.$$

The sensing SNR is given by

(31) $$\rho_{s}=\frac{E\;\left[{∥G[n,m]∥}_{F}^{2}\right]}{E\;\left[{∥\bar{N}\left[n,m\right]∥}_{F}^{2}\right]}.$$

The corresponding sensing SNR in decibels is given by ${\mathrm{SNR}}_{s,\mathrm{dB}}=10\log_{10}\rho_{s}$.

### 2.3. Model Assumptions

To provide a unified basis for subsequent sensing-signal processing, virtual-array construction, and angle estimation, the following model assumptions are adopted.

1. **Far-field co-located geometry assumption:** A co-located far-field sensing scenario is considered. The targets are assumed to be located in the far field of the array, such that the wavefront across the array aperture can be approximated as a plane wave. For the same target, the departure angle with respect to the transmit array and the arrival angle with respect to the receive array are approximately identical, and a common angular parameter is adopted to characterize its spatial direction.
2. **Point-target assumption:** Each sensing target is modeled as a point target whose echo is characterized by a complex scattering coefficient, propagation delay, Doppler frequency shift, and angular parameter, while the extended scattering structure of the target is neglected.
3. **Quasi-static platform and CPI-stationarity assumption:** The UAV is assumed to remain hovering or quasi-static within one CPI, with the target delay, Doppler frequency shift, angle, and complex scattering coefficient remaining approximately constant, while platform-motion-induced Doppler spreading, clutter ridge, and advanced motion-clutter processing such as STAP are not explicitly considered.
4. **Narrowband array-response assumption:** The narrowband array-response model is adopted, and the variation in the spatial-array response with OFDM subcarrier frequency is neglected. A common steering vector constructed using the carrier wavelength is employed for all subcarriers, while the frequency-dependent beam-squint effect is not considered.
5. **Array-coherence and ideal-calibration assumption:** The transmit and receive channels are assumed to remain phase coherent, with known antenna-element positions and relative phase relationships, while synchronization errors, array amplitude/phase mismatches, and hardware calibration errors are neglected.
6. **Noise statistical assumption:** The receiver noise is modeled as zero-mean circularly symmetric complex Gaussian noise, and the equivalent noise components of different transmit–receive channels are assumed to be mutually independent with the same variance $\sigma_{g}^{2}$.
7. **Self-interference suppression assumption:** The sensing-signal model is established based on the baseband received signal after self-interference cancellation (SIC), and the impact of residual self-interference is neglected.

## 3. Proposed Virtual-Array Configuration

**Figure 2:**
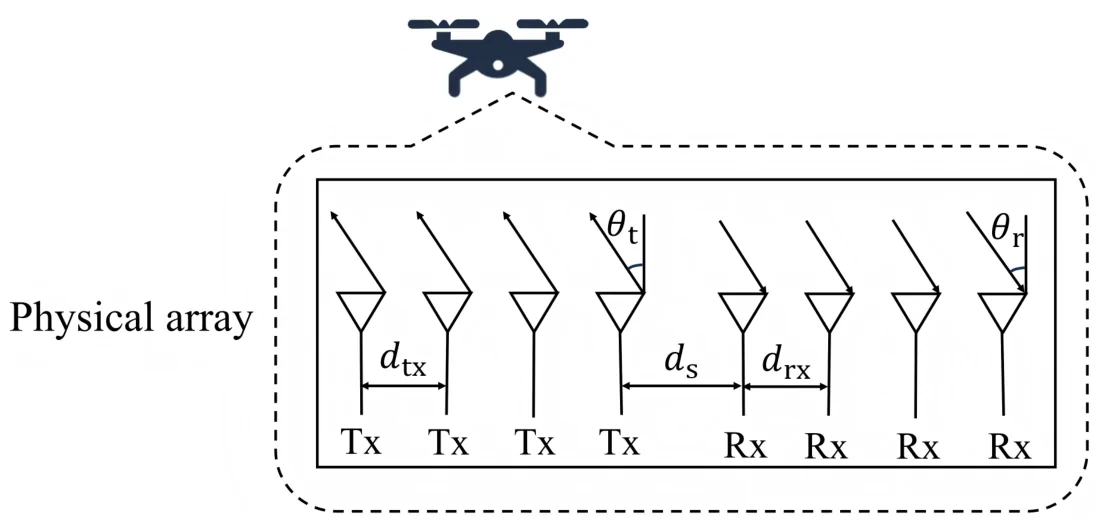
Physical geometry of the UAV-borne co-located MIMO array. An $N_{t}$-element transmit ULA and an $N_{r}$-element receive ULA are installed on the same UAV platform. The inter-element spacings of the transmit and receive arrays are $d_{\mathrm{tx}}$ and $d_{\mathrm{rx}}$, respectively, while the physical separation between the Tx and Rx arrays is denoted by $d_{s}$. For the monostatic configuration, the transmit and receive look angles satisfy $\theta_{t}=\theta_{r}=\theta$.

Due to UAV payload and installation-space constraints, the physical-array aperture and number of RF chains are limited. As shown in Figure 2, the transmitter and receiver employ co-located ULAs with $N_{t}$ and $N_{r}$ elements, respectively. The limited aperture restricts the resolution of closely spaced targets. Therefore, a virtual array is constructed from the transmit–receive MIMO channels to enlarge the effective aperture and improve angular resolution without adding physical antennas or RF chains.

### 3.1. Principle of Virtual-Array Construction

Orthogonal pilot-matched filtering yields the separated MIMO sensing-channel observation $\hat{G}\left[n,m\right]\in C^{N_{r}\times N_{t}}$.

We vectorize the sensing channel matrix and define the virtual-array observation as

(32) $$y_{s}\left[n,m\right]=\mathrm{vec}\left(\hat{G}\left[n,m\right]\right),$$

where $y_{s}\left[n,m\right]\in C^{N_{t}N_{r}\times 1}$, with each entry corresponding to one candidate virtual spatial sample associated with a transmit–receive antenna pair.

Using the separated sensing-channel observation $\hat{G}\left[n,m\right]$ obtained in (29), its vectorized form can be expanded as

(33) $$\begin{array}{cc} y_{s}\left[n,m\right] & =\mathrm{vec}\left(\hat{G}\left[n,m\right]\right)\\ \; & =\sum_{k=1}^{K}\alpha_{k}e^{-j2\pi n\Delta f\tau_{k}}e^{j2\pi f_{D,k}mT_{\mathrm{sym}}}\left(a_{\mathrm{tx}}\left(\theta_{k}\right)⊗a_{\mathrm{rx}}\left(\phi_{k}\right)\right)+c_{s}\left[n,m\right]+n_{s}\left[n,m\right], \end{array}$$

where $c_{s}\left[n,m\right]=\mathrm{vec}\;\left(C[n,m]\right)$ denotes the equivalent clutter and non-target interference term, $n_{s}\left[n,m\right]=\mathrm{vec}\;\left(\bar{N}\left[n,m\right]\right)$ denotes the equivalent noise term in the candidate virtual-array domain, and ⊗ denotes the Kronecker product.

The corresponding candidate virtual-array steering vector is defined as

(34) $$a_{v}\left(\theta_{k},\phi_{k}\right)≜a_{\mathrm{tx}}\left(\theta_{k}\right)⊗a_{\mathrm{rx}}\left(\phi_{k}\right),$$

whose dimension is $N_{t}N_{r}$, corresponding to the set of candidate virtual spatial channels formed by all transmit–receive antenna pairs.

Under the co-located far-field assumption in (20), the virtual steering vector becomes

(35) $$a_{v}\left(\vartheta_{k}\right)=a_{\mathrm{tx}}\left(\vartheta_{k}\right)⊗a_{\mathrm{rx}}\left(\vartheta_{k}\right),$$

and the candidate virtual-array observation model becomes

(36) $$y_{s}\left[n,m\right]=\sum_{k=1}^{K}\alpha_{k}e^{-j2\pi n\Delta f\tau_{k}}e^{j2\pi f_{D,k}mT_{\mathrm{sym}}}a_{v}\left(\vartheta_{k}\right)+c_{s}\left[n,m\right]+n_{s}\left[n,m\right].$$

The above derivation shows that, under a co-located monostatic MIMO configuration, the vectorized sensing channel observation can be naturally interpreted as a candidate virtual-array snapshot. In other words, the virtual array is not introduced as an additional assumption, but rather arises from the joint response of the physical transmit and receive arrays through vectorization.

Geometrically, each transmit–receive antenna pair corresponds to one candidate virtual element.

Let the transmit and receive element-position sets be $P_{t}=\left\{p_{t,1},p_{t,2},\ldots ,p_{t,N_{t}}\right\}$ and $P_{r}=\left\{p_{r,1},p_{r,2},\ldots ,p_{r,N_{r}}\right\}$, respectively.

Under a co-located MIMO configuration, each transmit–receive antenna pair corresponds to a virtual element whose equivalent spatial location is defined as

(37) $$p_{v,i,p}=p_{t,i}+p_{r,p},\;i=1,\ldots ,N_{t},\;p=1,\ldots ,N_{r}.$$

Accordingly, the $N_{t}N_{r}$ transmit–receive antenna pairs generate $N_{t}N_{r}$ candidate virtual spatial samples. The set of distinct virtual element locations is expressed as

(38) $$P_{v}=\left\{p_{t,i}+p_{r,p}\;|\;i=1,\ldots ,N_{t},\;p=1,\ldots ,N_{r}\right\}.$$

whose cardinality is given by

(39) $$Q=|P_{v}|\leq N_{t}N_{r},$$

Here, $N_{t}N_{r}$ represents the number of candidate virtual spatial samples generated by all transmit–receive antenna pairs, whereas *Q* denotes the number of distinct virtual-element locations. Due to the array geometry, different transmit–receive antenna pairs may map to the same spatial location. Therefore, *Q* can be smaller than $N_{t}N_{r}$, and the final virtual-array structure depends jointly on the transmit- and receive-array geometries.

### 3.2. Geometry-Aware Virtual-Array Construction

Under a uniform transmit- and receive-array configuration, a representative parameter setting is considered, where both the transmit and receive arrays consist of four elements with inter-element spacings given by

(40) $$d_{\mathrm{tx}}=d_{\mathrm{rx}}=d=\frac{\lambda }{2}.$$

For the considered uniform array configuration, the candidate virtual-element positions can be expressed as

(41) $$p_{v,i,p}=\left(i-1\right)\frac{\lambda }{2}+\left(p-1\right)\frac{\lambda }{2}=\left(i+p-2\right)d,\;i,p=1,\ldots ,4.$$

Since the virtual locations depend only on i+p, different transmit–receive pairs overlap. For i,p∈{1,2,3,4}, removing duplicate locations gives

(42) $$P_{v}^{\left(7\right)}=\left\{0,d,2d,3d,4d,5d,6d\right\},\;d=\frac{\lambda }{2}.$$

This set contains seven distinct virtual elements. The resulting virtual aperture length is, therefore, given by

(43) $$D^{\left(7\right)}=6d=3\lambda .$$

**Figure 3:**
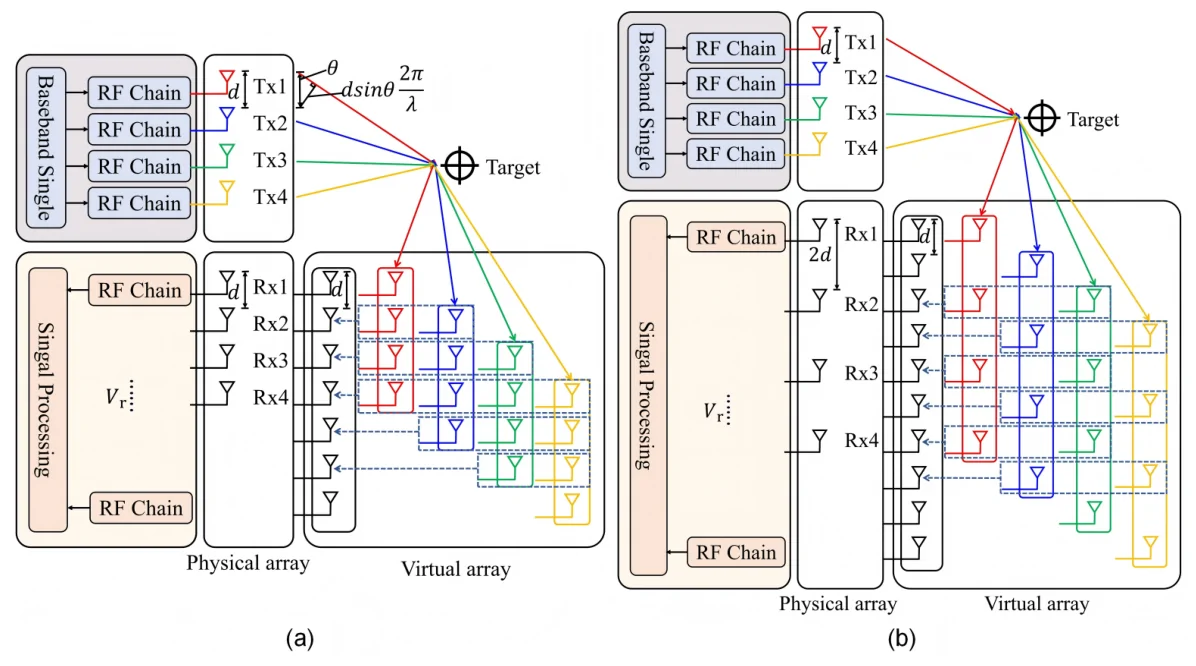
Geometric illustration of virtual-element formation under different transmit–receive array spacings. The red, blue, green, and yellow virtual elements correspond to the virtual array elements generated by Tx1, Tx2, Tx3, and Tx4, respectively, through their pairings with the receive-array elements. (**a**) Equal transmit- and receive-array spacings with $d_{\mathrm{tx}}=d_{\mathrm{rx}}=\lambda /2$, resulting in a virtual array with 7 distinct elements. (**b**) Unequal transmit- and receive-array spacings with $d_{\mathrm{tx}}=\lambda /2$ and $d_{\mathrm{rx}}=2d=\lambda$, resulting in a virtual array with 10 distinct elements.

Figure 3a illustrates the geometric formation of the virtual elements under the equal transmit–receive spacing configuration, i.e., $d_{\mathrm{tx}}=d_{\mathrm{rx}}=\lambda /2$. As shown in the Figure, different transmit–receive element pairings are mapped onto overlapping virtual-element locations in space, which leads to the formation of a virtual array with only seven distinct element positions, despite the fact that the total number of transmit–receive pairings is $N_{t}N_{r}=16$.

Building upon the Virtual-7 construction, we further investigate how to increase the number of distinct virtual elements and extend the effective virtual aperture by reducing the overlap among candidate virtual positions through transmit–receive spacing design, while keeping the number of physical antennas unchanged and maintaining a relatively compact physical array. The motivation for extending the virtual array from seven to ten elements is twofold: (i) more unique virtual sensor locations provide additional spatial samples and spatial degrees of freedom, and (ii) the increased effective aperture offers higher angular-resolution potential for subsequent angle estimation and multi-target discrimination.

Specifically, we set

(44) $$d_{\mathrm{tx}}=\frac{\lambda }{2},\;d_{\mathrm{rx}}=\lambda .$$

The positions of the new candidate virtual elements can be expressed as

(45) $$p_{v,i,p}=\left(i-1\right)\frac{\lambda }{2}+\left(p-1\right)\lambda =\left(i+2p-3\right)\frac{\lambda }{2},\;i,p=1,\ldots ,4.$$

Unlike the equal-spacing baseline where the virtual locations depend on the index combination i+p, the above expression shows that the mapping is governed by the spacing ratio between the transmit and receive arrays. For the considered spacing ratio, the spatial mapping of the transmit–receive pairs is reshaped, thereby reducing the overlap among the candidate virtual-element locations.

For i,p∈{1,2,3,4}, removing duplicate locations gives

(46) $$P_{v}^{\left(10\right)}=\left\{0,\frac{\lambda }{2},\lambda ,\ldots ,\frac{9\lambda }{2}\right\},$$

which contains 10 distinct virtual elements, corresponding to the Virtual-10 configuration. The effective aperture is, therefore, extended to

(47) $$D^{\left(10\right)}=\frac{9\lambda }{2}=4.5\lambda .$$

For the considered $N_{t}=N_{r}=4$ MIMO configuration, the $N_{t}N_{r}=16$ transmit–receive antenna pairs can generate up to 16 distinct virtual-element locations through an appropriate choice of transmit and receive array spacings. For example, when $d_{\mathrm{tx}}=\lambda /2$ and $d_{\mathrm{rx}}=2\lambda$, 16 uniformly distributed virtual elements can be formed, yielding an effective virtual aperture of 7.5λ, consistent with the classical MIMO virtual-array construction reported in [11,12].

It should be noted that the Virtual-10 configuration is not intended to maximize the number of distinct virtual elements. Although the above 16-element configuration provides a larger virtual aperture, it requires a receive-array physical aperture of 6λ. In comparison, the Virtual-10 configuration considered in this work uses $d_{\mathrm{rx}}=\lambda$ and requires only a 3λ receive-array aperture. Considering the installation-space constraints of UAV-mounted arrays, Virtual-10 is adopted as a representative compact configuration to investigate the trade-off between physical-array size and virtual-aperture enhancement.

Figure 3b illustrates the formation of the virtual elements under the unequal transmit–receive spacing configurations. By increasing the receive-array spacing to $d_{\mathrm{rx}}=2d=\lambda$, the transmit–receive pairwise mapping becomes more diversified, reducing the overlap among virtual-element locations.

### 3.3. Virtual-Array Data Construction

To clarify the data arrangement, we adopt the standard column-major vectorization convention for stacking the MIMO channel observations. Specifically, the elements in $y_{s}\left[n,m\right]$ are ordered such that the receive-antenna index *p* varies first and the transmit-antenna index *i* varies second. Under this column-major ordering, the vector index associated with the transmit–receive antenna pair (i,p) is given by

(48) $$r\left(i,p\right)=p+\left(i-1\right)N_{r},$$

where $r\left(i,p\right)\in \left\{1,\ldots ,N_{t}N_{r}\right\}$. Let ${\hat{g}}_{i,p}\left[n,m\right]$ denote the separately recovered channel observation associated with the *i*th transmit antenna and the *p*th receive antenna, i.e., ${\hat{g}}_{i,p}\left[n,m\right]={\left[\hat{G}\left[n,m\right]\right]}_{p,i}$.

According to (37), define the virtual-position index q(i,p) to map each transmit–receive pair to its corresponding distinct virtual location

(49) q(i,p)∈{1,2,…,Q},

where q(i,p) denotes the index of the distinct virtual-element location associated with the pair (i,p). For the Virtual-7 and Virtual-10 configurations considered in this work, Q=7 and Q=10, respectively.

We further define the virtual-array mapping matrix $T_{v}\in C^{Q\times N_{t}N_{r}}$, which maps the candidate virtual observation to the final virtual-array snapshot:

(50) $$y_{v}\left[n,m\right]=T_{v}y_{s}\left[n,m\right],$$

where $y_{v}\left[n,m\right]\in C^{Q\times 1}$ is the merged virtual-array observation before noise whitening.

Each column of $T_{v}$ corresponds to a transmit–receive pair and each row to a distinct virtual location. For pair (i,p), the entry at row q(i,p) and column r(i,p) is assigned weight $\omega_{i,p}$, while the remaining entries are zero:

(51) $${\left[T_{v}\right]}_{q,r(i,p)}=\left\{\begin{array}{cc} \omega_{i,p}, & q=q(i,p),\\ 0, & q\neq q(i,p). \end{array}\right.$$

When multiple transmit–receive element pairs are mapped to the same virtual position, their observations are merged into the same virtual element. Let $S_{q}=\left\{\left(i,p\right)|q\left(i,p\right)=q\right\}$ denote the set of transmit–receive pairs associated with the *q*th virtual element. Then, the observation of the *q*th virtual element is given by

(52) $$y_{v}\left(q;n,m\right)=\sum_{\left(i,p\right)\in S_{q}}\omega_{i,p}{\hat{g}}_{i,p}\left[n,m\right].$$

Equal-weight averaging is adopted for duplicated virtual elements, namely,

(53) $$\omega_{i,p}=\frac{1}{|S_{q}|},\;\left(i,p\right)\in S_{q}.$$

For example, in Virtual-7, the pairs (1,3), (2,2), and (3,1) all map to the virtual location 2d and are, therefore, merged. In Virtual-10, the different transmit and receive spacings reduce such overlap and yield more distinct virtual elements.

After equal-weight averaging, the noise variance of the *q*th virtual element is $\sigma_{g}^{2}/\left|S_{q}\right|$. Accordingly, define the redundancy matrix as

(54) $$D=\mathrm{diag}\left(|S_{1}\left|,\right|S_{2}\left|,\ldots ,\right|S_{Q}|\right).$$

The corresponding noise covariance matrix is

(55) $$R_{n,v}=\sigma_{g}^{2}D^{-1}.$$

The noise-whitened virtual-array observation is then obtained as

(56) $${\tilde{y}}_{v}\left[n,m\right]=D^{\frac{1}{2}}y_{v}\left[n,m\right].$$

The multiplication by $D^{1/2}$ compensates for the redundancy-dependent noise variance introduced by equal-weight averaging. Consequently, the noise covariance matrix of the whitened virtual-array observation becomes

(57) $$R_{\tilde{n},v}=D^{\frac{1}{2}}R_{n,v}D^{\frac{1}{2}}=\sigma_{g}^{2}I_{Q}.$$

The covariance matrix of the whitened virtual-array observation is

(58) $$R_{v}=E\left[{\tilde{y}}_{v}\left[n,m\right]{\tilde{y}}_{v}^{H}\left[n,m\right]\right].$$

This matrix is used for subsequent angle processing and spatial-spectrum estimation.

## 4. Signal Processing with Physical and Virtual Arrays

### 4.1. Range–Doppler Processing

After obtaining the time–frequency array observations, radar data cubes are constructed for range–Doppler processing. To describe the physical array, Virtual-7, and Virtual-10 within a unified framework, define

(59) χ∈{phy,v7,v10},

where phy, v7, and v10 denote the physical array, Virtual-7 array, and Virtual-10 array, respectively. The corresponding number of spatial samples is denoted by $M_{\chi }$, with $M_{\mathrm{phy}}=N_{r}$, $M_{v7}=7$, and $M_{v10}=10$.

For physical-array processing, the transmit-separated sensing-channel observations are retained individually. Specifically, the observation associated with the *i*th transmit antenna is defined as

(60) $$g_{\mathrm{phy},i}\left[n,m\right]=\hat{G}\left[n,m\right]e_{i},\;i=1,\ldots ,N_{t},$$

where $e_{i}\in C^{N_{t}\times 1}$ denotes the *i*th canonical basis vector. Hence, $g_{\mathrm{phy},i}\left[n,m\right]\in C^{N_{r}\times 1}$ contains the observations of the $N_{r}$ physical receive antennas associated with the *i*th separated transmit channel. All $N_{t}$ transmit-separated observations are retained for subsequent range–Doppler processing.

For the physical array, the $N_{t}$ transmit-separated observations $g_{\mathrm{phy},i}\left[n,m\right]$, $i=1,\ldots ,N_{t}$, are processed individually along the subcarrier and OFDM-symbol dimensions.

For the virtual arrays, the array-domain observation is defined as

(61) $$x_{\chi }\left[n,m\right]=\left\{\begin{array}{cc} {\tilde{y}}_{v}^{\left(7\right)}\left[n,m\right],\; & \chi =v7,\\ {\tilde{y}}_{v}^{\left(10\right)}\left[n,m\right],\; & \chi =v10, \end{array}\right.$$

where $x_{\chi }\left[n,m\right]\in C^{M_{\chi }\times 1}$. Here, ${\tilde{y}}_{v}^{\left(7\right)}\left[n,m\right]$ and ${\tilde{y}}_{v}^{\left(10\right)}\left[n,m\right]$ denote the virtual-array observations after virtual mapping, duplicate-position merging, and noise whitening, respectively.

For the physical array, a separate radar data cube is constructed for each transmit-separated observation by stacking $g_{\mathrm{phy},i}\left[n,m\right]$ over the subcarrier and OFDM-symbol dimensions, such that $X_{\mathrm{phy}}^{\left(i\right)}\in C^{N_{r}\times N_{c}\times N_{s}}$, $i=1,\ldots ,N_{t}$, where the three dimensions correspond to the physical receive antennas, subcarriers, and OFDM symbols, respectively.

For the virtual arrays, $X_{\chi }\in C^{M_{\chi }\times N_{c}\times N_{s}}$, χ∈{v7,v10}, with dimensions corresponding to virtual elements, subcarriers, and OFDM symbols, respectively.

Range processing is first performed along the subcarrier dimension.

For the physical array, the range IFFT is independently applied to each transmit-separated radar data cube as

(62) $$Z_{\mathrm{phy}}^{\left(i\right)}\left(q,r,m\right)=\frac{1}{N_{c}}\sum_{n=0}^{N_{c}-1}X_{\mathrm{phy}}^{\left(i\right)}\left(q,n,m\right)e^{j2\pi \frac{nr}{N_{c}}},$$

where $q=1,\ldots ,N_{r}$, $i=1,\ldots ,N_{t}$, $r=0,\ldots ,N_{c}-1$, and $m=0,\ldots ,N_{s}-1$. The resulting range-processed physical-array data cube satisfies $Z_{\mathrm{phy}}^{\left(i\right)}\in C^{N_{r}\times N_{c}\times N_{s}}$.

For the virtual arrays, range processing is performed as

(63) $$Z_{\chi }\left(q,r,m\right)=\frac{1}{N_{c}}\sum_{n=0}^{N_{c}-1}X_{\chi }\left(q,n,m\right)e^{j2\pi \frac{nr}{N_{c}}},\;\chi \in \left\{v7,v10\right\},$$

where $q=1,\ldots ,M_{\chi }$, $r=0,\ldots ,N_{c}-1$, and $m=0,\ldots ,N_{s}-1$. The resulting range-processed virtual-array data cube satisfies $Z_{\chi }\in C^{M_{\chi }\times N_{c}\times N_{s}}$.

After range processing, Doppler processing is performed along the OFDM- symbol dimension.

For the physical array, Doppler processing is independently applied to each transmit-separated range-processed data cube as

(64) $$D_{\mathrm{phy}}^{\left(i\right)}\left(q,r,d\right)=\sum_{m=0}^{N_{s}-1}Z_{\mathrm{phy}}^{\left(i\right)}\left(q,r,m\right)e^{-j2\pi \frac{md}{N_{s}}},$$

where $q=1,\ldots ,N_{r}$, $i=1,\ldots ,N_{t}$, and $d=0,\ldots ,N_{s}-1$. Hence, $D_{\mathrm{phy}}^{\left(i\right)}\in C^{N_{r}\times N_{c}\times N_{s}}$.

For the virtual arrays, Doppler processing is performed as

(65) $$D_{\chi }\left(q,r,d\right)=\sum_{m=0}^{N_{s}-1}Z_{\chi }\left(q,r,m\right)e^{-j2\pi \frac{md}{N_{s}}},\;\chi \in \left\{v7,v10\right\},$$

where $d=0,\ldots ,N_{s}-1$. The resulting virtual-array range–Doppler data cube satisfies $D_{\chi }\in C^{M_{\chi }\times N_{c}\times N_{s}}$.

For physical-array range–Doppler map generation, the contributions of the $N_{t}$ transmit-separated channels are combined noncoherently in the power domain as

(66) $$P_{\mathrm{RD},\mathrm{phy}}\left(r,d\right)=\frac{1}{N_{t}}\sum_{i=1}^{N_{t}}{∥D_{\mathrm{phy}}^{\left(i\right)}\left(:,r,d\right)∥}_{2}^{2}.$$

This power-domain combination exploits all $N_{t}N_{r}$ separated transmit–receive channel measurements while preserving the spatial aperture of the $N_{r}$ physical receive antennas.

For the virtual arrays, the range–Doppler map is obtained by combining the virtual spatial-element powers as

(67) $$P_{\mathrm{RD},\chi }\left(r,d\right)={∥D_{\chi }\left(:,r,d\right)∥}_{2}^{2},\;\chi \in \left\{v7,v10\right\}.$$

For range–Doppler map visualization, the range-bin index and the Doppler-bin index are mapped to physical range and Doppler frequency, respectively. The range corresponding to the *r*th range bin is

(68) $$R_{r}=\frac{c_{0}r}{2N_{c}\Delta f},$$

where $c_{0}$ is the speed of light. After Doppler spectrum centering, the Doppler frequency corresponding to the *d*th Doppler bin is

(69) $$f_{D,d}=\frac{d-\frac{N_{s}}{2}}{N_{s}T_{\mathrm{sym}}},\;d=0,\ldots ,N_{s}-1.$$

### 4.2. Angle Processing with Physical and Virtual Arrays

#### 4.2.1. Spatial Snapshot and Steering-Vector Construction

After range–Doppler processing, the spatial-domain observations at a selected range–Doppler bin are extracted for subsequent angle estimation.

For the physical array, the spatial observation of the *i*th transmit-separated channel is defined as

(70) $$u_{\mathrm{phy},i}\left(r,d\right)=D_{\mathrm{phy}}^{\left(i\right)}\left(:,r,d\right)\in C^{N_{r}\times 1},\;i=1,\ldots ,N_{t}.$$

The vector $u_{\mathrm{phy},i}\left(r,d\right)$ contains the observations of the $N_{r}$ physical receive antennas, and all $N_{t}$ transmit-separated channels are retained.

For the virtual arrays, the spatial observation is defined as

(71) $$u_{\chi }\left(r,d\right)=D_{\chi }\left(:,r,d\right)\in C^{M_{\chi }\times 1},\;\chi \in \left\{v7,v10\right\}.$$

For the Virtual-7 and Virtual-10 arrays, the entries of $u_{v7}\left(r,d\right)$ and $u_{v10}\left(r,d\right)$ correspond to the unique virtual elements, respectively.

To describe different array configurations in a unified form, the spatial position set of configuration χ is denoted by

(72) $$P_{\chi }=\left\{p_{\chi ,1},p_{\chi ,2},\ldots ,p_{\chi ,M_{\chi }}\right\},$$

where $p_{\chi ,q}$ represents the position of the *q*th spatial element. These positions correspond to the physical receive-antenna locations for the physical array and to the unique virtual-element locations for the virtual arrays.

Based on $P_{\chi }$, the steering vector for a candidate angle ϑ is constructed as

(73) $$a_{\chi }\left(\vartheta \right)={\left[e^{-j\frac{2\pi }{\lambda }p_{\chi ,1}\sin\vartheta },e^{-j\frac{2\pi }{\lambda }p_{\chi ,2}\sin\vartheta },\ldots ,e^{-j\frac{2\pi }{\lambda }p_{\chi ,M_{\chi }}\sin\vartheta }\right]}^{T},$$

For the virtual arrays, since the array observations have been transformed by the redundancy-dependent whitening matrix, the steering vector used for angle processing must undergo the same transformation to maintain consistency with the observation model. Accordingly, the effective steering vector is defined as

(74) $${\bar{a}}_{\chi }\left(\vartheta \right)=\left\{\begin{array}{cc} a_{\chi }\left(\vartheta \right),\; & \chi =\mathrm{phy},\\ D_{\chi }^{\frac{1}{2}}a_{\chi }\left(\vartheta \right),\; & \chi \in \{v7,v10\}, \end{array}\right.$$

where $D_{\chi }$ denotes the redundancy matrix corresponding to the considered virtual-array configuration. For the physical array, $a_{\chi }\left(\vartheta \right)$ is used with the $N_{t}$ transmit-separated physical receive-array observations. For the virtual arrays, the effective steering vector ${\bar{a}}_{\chi }\left(\vartheta \right)$ is used together with the corresponding noise-whitened virtual-array observation for subsequent angle processing.

#### 4.2.2. Conventional Beamforming-Based Angle Processing

After obtaining the spatial observations at the selected range–Doppler bin, conventional beamforming (CBF) is applied for angle scanning according to the corresponding physical- or virtual-array observation model.

For the physical array, CBF is independently applied to the $N_{t}$ transmit-separated observations and the resulting powers are combined as

(75) $$P_{\mathrm{CBF},\mathrm{phy}}\left(\vartheta ;r,d\right)=\frac{1}{N_{t}}\sum_{i=1}^{N_{t}}\frac{{\left|a_{\mathrm{phy}}^{H}\left(\vartheta \right)u_{\mathrm{phy},i}\left(r,d\right)\right|}^{2}}{a_{\mathrm{phy}}^{H}\left(\vartheta \right)a_{\mathrm{phy}}\left(\vartheta \right)}.$$

For the virtual arrays, the CBF spatial spectrum is given by

(76) $$P_{\mathrm{CBF},\chi }\left(\vartheta ;r,d\right)=\frac{{\left|{\bar{a}}_{\chi }^{H}\left(\vartheta \right)u_{\chi }\left(r,d\right)\right|}^{2}}{{\bar{a}}_{\chi }^{H}\left(\vartheta \right){\bar{a}}_{\chi }\left(\vartheta \right)},\;\chi \in \left\{v7,v10\right\}.$$

By scanning the candidate angle set Θ, the CBF-based angle estimate is obtained as

(77) $${\hat{\vartheta }}_{\mathrm{CBF},\chi }\left(r,d\right)=\arg\max_{\vartheta \in \Theta }P_{\mathrm{CBF},\chi }\left(\vartheta ;r,d\right).$$

For multiple detected targets, CBF angle scanning can be performed at the corresponding detected range–Doppler bins, and the dominant peak of each spatial spectrum provides the associated angle estimate.

CBF does not require covariance-matrix decomposition, but its angular resolution is limited by the array aperture and mainlobe width. Therefore, MUSIC is introduced in the following subsection for high-resolution angle estimation.

#### 4.2.3. MUSIC-Based High-Resolution Angle Processing

To improve angular resolution, MUSIC exploits the subspace structure of the spatial covariance matrix. For the MUSIC-based DOA evaluation, the common sensing and array models established above are specialized to the spatial angular dimension. Let $\ell_{s}=1,\ldots ,L_{s}$ denote the index of the repeated sensing acquisitions used for covariance estimation. Each sensing acquisition contains the complete $L_{o}$ orthogonal pilot dimensions and, after transmit-pilot-matched filtering, yields a transmit-separated sensing-channel observation matrix ${\hat{G}}^{\left(\ell_{s}\right)}\in C^{N_{r}\times N_{t}}$, $\ell_{s}=1,\ldots ,L_{s}$.

Let $\gamma^{\left(\ell_{s}\right)}={\left[\gamma_{1}^{\left(\ell_{s}\right)},\ldots ,\gamma_{K}^{\left(\ell_{s}\right)}\right]}^{T}$ denote the effective complex target coefficients in the $\ell_{s}$th sensing acquisition.

For the physical array, all $N_{t}$ transmit-separated observations $g_{\mathrm{phy},i}^{\left(\ell_{s}\right)}={\hat{G}}^{\left(\ell_{s}\right)}e_{i}$, $i=1,\ldots ,N_{t}$, are retained for spatial covariance estimation. The contribution of the *k*th target to the *i*th transmit-separated physical-array observation is proportional to $\gamma_{k}^{\left(\ell_{s}\right)}{\left[a_{\mathrm{tx}}\left(\vartheta_{k}\right)\right]}_{i}a_{\mathrm{phy}}\left(\vartheta_{k}\right)$.

For the virtual arrays, the same transmit-separated observation is processed by virtual mapping, duplicate-position merging, and noise whitening to obtain the spatial snapshot $v_{\chi }^{\left(\ell_{s}\right)}\in C^{M_{\chi }\times 1}$, χ∈{v7,v10}.

The corresponding virtual-array angle-domain observation model is expressed as

(78) $$v_{\chi }^{\left(\ell_{s}\right)}={\bar{A}}_{\chi }\left(\vartheta \right)\gamma^{\left(\ell_{s}\right)}+n_{\chi }^{\left(\ell_{s}\right)},\;\chi \in \left\{v7,v10\right\},$$

where ${\bar{A}}_{\chi }\left(\vartheta \right)=\left[{\bar{a}}_{\chi }\left(\vartheta_{1}\right),\ldots ,{\bar{a}}_{\chi }\left(\vartheta_{K}\right)\right]$ is the effective virtual-array manifold matrix, and $n_{\chi }^{\left(\ell_{s}\right)}$ denotes the corresponding spatial noise vector.

For the MUSIC-based angle-domain evaluation, the effective complex coefficients associated with different targets are modeled as statistically uncorrelated, i.e., $E[\gamma_{k}^{\left(\ell_{s}\right)}\gamma_{k^{\prime }}^{(\ell_{s})*}]=0$ for $k\neq k^{\prime }$.

For the physical array, the receive-array covariance matrix is constructed using all $N_{t}$ transmit-separated observations. Its theoretical covariance matrix is expressed as

(79) $$R_{\mathrm{phy}}=\frac{1}{N_{t}}\sum_{i=1}^{N_{t}}E\left[g_{\mathrm{phy},i}g_{\mathrm{phy},i}^{H}\right]=\frac{1}{N_{t}}E\left[\hat{G}{\hat{G}}^{H}\right].$$

In practice, the physical-array covariance matrix is estimated from the $L_{s}$ sensing acquisitions as

(80) $${\hat{R}}_{\mathrm{phy}}=\frac{1}{N_{t}L_{s}}\sum_{\ell_{s}=1}^{L_{s}}\sum_{i=1}^{N_{t}}g_{\mathrm{phy},i}^{\left(\ell_{s}\right)}{\left(g_{\mathrm{phy},i}^{\left(\ell_{s}\right)}\right)}^{H}=\frac{1}{N_{t}L_{s}}\sum_{\ell_{s}=1}^{L_{s}}{\hat{G}}^{\left(\ell_{s}\right)}{\left({\hat{G}}^{\left(\ell_{s}\right)}\right)}^{H}.$$

Therefore, ${\hat{R}}_{\mathrm{phy}}\in C^{N_{r}\times N_{r}}$.

For the virtual arrays, the spatial covariance matrix is expressed as

(81) $$R_{\chi }=E\left[v_{\chi }^{\left(\ell_{s}\right)}{\left(v_{\chi }^{\left(\ell_{s}\right)}\right)}^{H}\right],\;\chi \in \left\{v7,v10\right\}.$$

The corresponding sample covariance matrix is estimated as

(82) $${\hat{R}}_{\chi }=\frac{1}{L_{s}}\sum_{\ell_{s}=1}^{L_{s}}v_{\chi }^{\left(\ell_{s}\right)}{\left(v_{\chi }^{\left(\ell_{s}\right)}\right)}^{H},\;\chi \in \left\{v7,v10\right\}.$$

Hence, ${\hat{R}}_{\mathrm{phy}}$, ${\hat{R}}_{v7}$, and ${\hat{R}}_{v10}$ have dimensions $N_{r}\times N_{r}$, 7×7, and 10×10, respectively, and are collectively denoted by ${\hat{R}}_{\chi }$ below.

The sample covariance matrix is then eigendecomposed as

(83) $${\hat{R}}_{\chi }=E_{s,\chi }\Lambda_{s,\chi }E_{s,\chi }^{H}+E_{n,\chi }\Lambda_{n,\chi }E_{n,\chi }^{H},$$

where the eigenvectors associated with the largest *K* eigenvalues form the signal subspace $E_{s,\chi }$, while the remaining eigenvectors form the noise subspace $E_{n,\chi }$, for $K<M_{\chi }$.

The MUSIC spatial spectrum is constructed by exploiting the orthogonality between the steering vector and the noise subspace. Using the effective steering vector ${\bar{a}}_{\chi }\left(\vartheta \right)$ defined in (74), the MUSIC spatial spectrum is given by

(84) $$P_{\mathrm{MUSIC},\chi }\left(\vartheta \right)=\frac{1}{{∥E_{n,\chi }^{H}{\bar{a}}_{\chi }\left(\vartheta \right)∥}_{2}^{2}}.$$

For a multi-target scenario with *K* targets, the estimated DOA vector, ${\hat{\vartheta }}_{\chi }={\left[{\hat{\vartheta }}_{\chi ,1},\ldots ,{\hat{\vartheta }}_{\chi ,K}\right]}^{T}$, is obtained from the *K* selected dominant local peaks of the MUSIC spectrum.

### 4.3. Performance Evaluation Metrics

To quantitatively compare the DOA-estimation performance of the physical array, the Virtual-7 array, and the Virtual-10 array, four evaluation metrics are adopted: DOA RMSE, root CRB, probability of resolution, and conditional DOA RMSE.

#### 4.3.1. DOA RMSE

Let *K* denote the number of targets and $N_{\mathrm{MC}}$ denote the number of Monte Carlo trials. For array configuration χ, the estimated DOA of the *k*th target in the *i*th trial is denoted by ${\hat{\vartheta }}_{\chi ,k}^{\left(i\right)}$, while the corresponding true angle is $\vartheta_{k}$. The DOA RMSE is defined as

(85) $${\mathrm{RMSE}}_{\chi }=\sqrt{\frac{1}{N_{\mathrm{MC}}K}\sum_{i=1}^{N_{\mathrm{MC}}}\sum_{k=1}^{K}{\left({\hat{\vartheta }}_{\chi ,k}^{\left(i\right)}-\vartheta_{k}\right)}^{2}}.$$

For multi-target scenarios, the estimated angles are first matched with the true angles according to the minimum angular-error criterion to avoid target-index permutation. The DOA RMSE reflects the average angular-estimation accuracy of array configuration χ. When the angles are expressed in degrees, the RMSE is also measured in degrees.

#### 4.3.2. Cramér–Rao Bound

Based on the spatial observation model and the theoretical covariance matrix $R_{\chi }$ defined above, the Cramér–Rao bound (CRB) is adopted to characterize the theoretical variance lower bound for the two-target DOA estimation. For the single-target case, a closed-form CRB derivation can be found in [31]. Here, the corresponding closed-form CRB is derived for the two-target DOA scenario considered in this work.

Assume that the complex coefficients of the two targets are statistically uncorrelated. The signal power of the *k*th target is defined as $p_{k}=E[|\gamma_{k}^{\left(\ell_{s}\right)}{|}^{2}]$, k=1,2.

For array configuration χ, let

(86) $${\bar{a}}_{\chi ,k}={\bar{a}}_{\chi }\left(\vartheta_{k}\right),\;{\dot{\bar{a}}}_{\chi ,k}=\frac{\partial {\bar{a}}_{\chi }\left(\vartheta_{k}\right)}{\partial \vartheta_{k}},\;k=1,2,$$

where ${\dot{\bar{a}}}_{\chi ,k}$ denotes the derivative of the effective steering vector with respect to the *k*th target DOA.

The derivative of the theoretical covariance matrix with respect to $\vartheta_{k}$ is then given by

(87) $$\frac{\partial R_{\chi }}{\partial \vartheta_{k}}=p_{k}\left({\dot{\bar{a}}}_{\chi ,k}{\bar{a}}_{\chi ,k}^{H}+{\bar{a}}_{\chi ,k}{\dot{\bar{a}}}_{\chi ,k}^{H}\right),\;k=1,2.$$

For $L_{s}$ independent spatial snapshots, the Fisher information matrix (FIM) associated with the two DOA parameters is expressed as

(88) $$J_{\chi }=\left[\begin{array}{cc} J_{\chi ,11} & J_{\chi ,12}\\ J_{\chi ,12} & J_{\chi ,22} \end{array}\right],$$

where $J_{\chi ,kk^{\prime }}$ denotes the $\left(k,k^{\prime }\right)$th element of the FIM and is given by

(89) Jχ,kk′=LsRetrRχ−1∂Rχ∂ϑkRχ−1∂Rχ∂ϑk′,k,k′∈{1,2}.

It can be seen that the array geometry of each configuration is incorporated into the FIM through the corresponding effective steering vectors ${\bar{a}}_{\chi ,k}$ and their angular derivatives ${\dot{\bar{a}}}_{\chi ,k}$.

For the above 2×2 FIM, its inverse can be explicitly written as

(90) $$J_{\chi }^{-1}=\frac{1}{J_{\chi ,11}J_{\chi ,22}-J_{\chi ,12}^{2}}\left[\begin{array}{cc} J_{\chi ,22} & -J_{\chi ,12}\\ -J_{\chi ,12} & J_{\chi ,11} \end{array}\right].$$

Accordingly, the closed-form CRBs of the two target DOAs are, respectively, given by

(91) $${\mathrm{CRB}}_{\chi }\left(\vartheta_{1}\right)=\frac{J_{\chi ,22}}{J_{\chi ,11}J_{\chi ,22}-J_{\chi ,12}^{2}},\;{\mathrm{CRB}}_{\chi }\left(\vartheta_{2}\right)=\frac{J_{\chi ,11}}{J_{\chi ,11}J_{\chi ,22}-J_{\chi ,12}^{2}}.$$

Here, $J_{\chi ,11}$ and $J_{\chi ,22}$ represent the Fisher information associated with the two DOA parameters, respectively, while the cross term $J_{\chi ,12}$ characterizes the coupling between them. As the angular separation between the two targets decreases, the correlation between their steering vectors generally increases, which strengthens the parameter coupling and consequently increases the corresponding CRBs.

The two-target root CRB is defined as

(92) $${\mathrm{RootCRB}}_{\chi }=\sqrt{\frac{{\mathrm{CRB}}_{\chi }\left(\vartheta_{1}\right)+{\mathrm{CRB}}_{\chi }\left(\vartheta_{2}\right)}{2}}.$$

When expressed in degrees, the root CRB is converted from radians by multiplying by 180/π.

Substituting the above closed-form CRBs yields

(93) $${\mathrm{RootCRB}}_{\chi }=\sqrt{\frac{J_{\chi ,11}+J_{\chi ,22}}{2\left(J_{\chi ,11}J_{\chi ,22}-J_{\chi ,12}^{2}\right)}}.$$

The above closed-form expression applies to Physical-4, Virtual-7, and Virtual-10, with the differences among the array configurations reflected in their corresponding effective steering vectors, angular derivatives, and theoretical covariance matrices.

For Virtual-7 and Virtual-10, the synthesized apertures are $D_{v7}=3\lambda$ and $D_{v10}=4.5\lambda$, respectively. Accordingly, the two-target root CRB for each array configuration is obtained by substituting its corresponding FIM elements into the above closed-form expression. Hence, the different virtual-array geometries and synthesized apertures are directly incorporated into their respective CRB calculations.

#### 4.3.3. Probability of Resolution

For array configuration χ, let ${\hat{\vartheta }}_{\chi ,k}^{\left(i\right)}$ denote the estimated DOA of the *k*th target in the *i*th Monte Carlo trial. The true angular separation and the estimated angular separation are defined as

(94) $$\Delta \vartheta =\left|\vartheta_{2}-\vartheta_{1}\right|,\;{\hat{\Delta \vartheta }}_{\chi }^{\left(i\right)}=\left|{\hat{\vartheta }}_{\chi ,2}^{\left(i\right)}-{\hat{\vartheta }}_{\chi ,1}^{\left(i\right)}\right|.$$

When two valid and distinct significant angular-spectrum peaks are detected in the *i*th trial, the association between the estimated peaks and the corresponding true targets is determined by

(95) $$\left|{\hat{\vartheta }}_{\chi ,k}^{\left(i\right)}-\vartheta_{k}\right|<\frac{\Delta \vartheta }{2},\;k=1,2.$$

In addition, the estimated angular separation is required to satisfy

(96) $$\left|{\hat{\Delta \vartheta }}_{\chi }^{\left(i\right)}-\Delta \vartheta \right|\leq \tau_{\Delta },$$

where $\tau_{\Delta }$ denotes the angular-separation error tolerance.

Accordingly, the successful-resolution indicator is defined as

(97) $$s_{\chi }^{\left(i\right)}=\left\{\begin{array}{cc} 1, & \mathrm{if}\;\mathrm{the}\;i\mathrm{th}\;\mathrm{trial}\;\mathrm{satisfies}\;\mathrm{the}\;\mathrm{above}\;\mathrm{resolution}\;\mathrm{conditions},\\ 0, & \mathrm{otherwise}. \end{array}\right.$$

The probability of resolution for array configuration χ is then defined as

(98) $$P_{r,\chi }=\frac{1}{N_{\mathrm{tot}}}\sum_{i=1}^{N_{\mathrm{tot}}}s_{\chi }^{\left(i\right)},$$

where $N_{\mathrm{tot}}$ denotes the total number of trials included in the statistical evaluation.

#### 4.3.4. Conditional DOA RMSE

For array configuration χ, the number of successfully resolved trials is defined as

(99) $$N_{\mathrm{res},\chi }=\sum_{i=1}^{N_{\mathrm{tot}}}s_{\chi }^{\left(i\right)}.$$

For $N_{\mathrm{res},\chi }>0$, the conditional DOA RMSE is defined as

(100) $${\mathrm{RMSE}}_{\mathrm{cond},\chi }=\sqrt{\frac{1}{N_{\mathrm{res},\chi }}\sum_{i=1}^{N_{\mathrm{tot}}}s_{\chi }^{\left(i\right)}\frac{1}{K}\sum_{k=1}^{K}{\left({\hat{\vartheta }}_{\chi ,k}^{\left(i\right)}-\vartheta_{k}\right)}^{2}},$$

where *K* denotes the number of targets involved in the DOA estimation. Only the trials satisfying $s_{\chi }^{\left(i\right)}=1$ are included in the calculation of the conditional DOA RMSE.

## 5. Simulation Results

### 5.1. Simulation Setup

Simulations are conducted for a UAV-assisted MIMO-OFDM ISAC system to verify the effectiveness of the proposed virtual-array method. The system adopts a co-located monostatic MIMO architecture, where a UAV equipped with a 4×4 array serves one ground-communication user while sensing multiple targets.

Three array configurations are compared: Physical-4, Virtual-7, and Virtual-10. Physical-4 denotes the physical receive array, while Virtual-7 and Virtual-10 denote the virtual arrays with seven and ten distinct virtual elements, respectively. For a fair comparison, the same communication model, sensing model, target scenario, and signal-processing procedure are used for all configurations, and the main difference lies in the effective aperture for angle-domain processing.

The communication performance is evaluated using the achievable rate, SINR, and the power-allocation factor α. The sensing performance is evaluated using range–Doppler maps, angle–Doppler maps, angular spectra, and statistical two-target DOA metrics. Unless otherwise specified, all simulation parameters are set according to Table 1. The physical Tx- and Rx-array element spacings and the Tx–Rx physical separation for the considered array configurations are summarized in Table 2.

### 5.2. Communication-Performance Evaluation

This subsection evaluates the communication performance of the considered UAV-ISAC system to verify that reliable data transmission can be maintained while supporting sensing functionality. In the communication simulation, the UAV acts as an aerial ISAC base station and hovers at (0,0,50)m, while the ground-communication user is fixed at (120,40,0)m. Meanwhile, multiple sensing targets are deployed within the sensing region in front of the UAV for target detection and angle estimation. The communication–sensing-power-allocation factor α is adopted to allocate the transmit power between the communication and sensing functions, where a fraction α of the transmit power is assigned to communication and the remaining (1−α) is allocated to sensing. Therefore, this subsection focuses on the impact of α on the communication performance to demonstrate that reliable communication can still be maintained while supporting sensing tasks.

**Figure 4:**
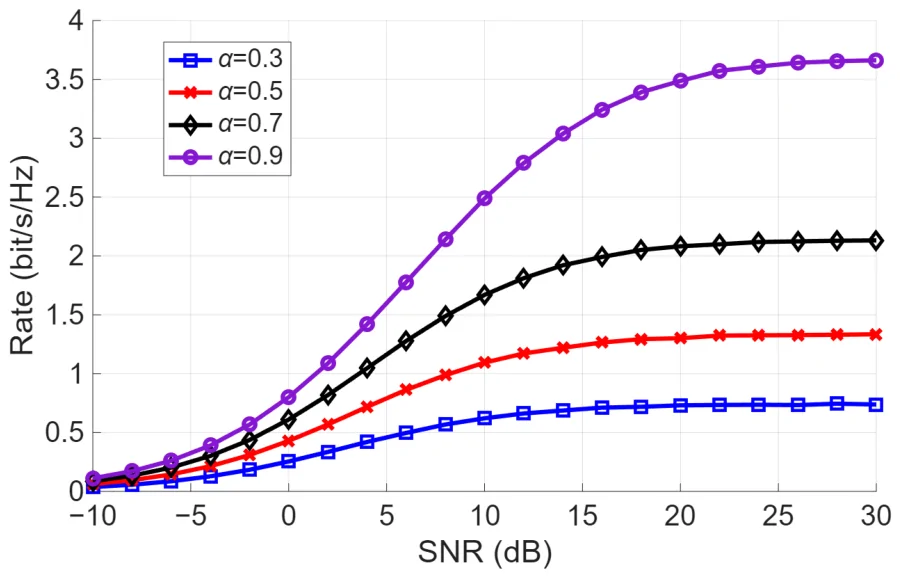
Achievable communication rate versus receive SNR under different communication–sensing-power-allocation factors α.

Figure 4 shows the achievable communication rate versus receive SNR for different values of α. The communication rate increases with receive SNR in all cases, while a larger α yields a higher rate because more transmit power is allocated to communication. Accordingly, α=0.9 achieves the highest rate, whereas α=0.3 gives the lowest.

These results demonstrate that α effectively controls the communication–sensing power tradeoff. The proposed virtual-array design enhances sensing capability without altering the physical communication link. By properly selecting α, sensing performance can be improved while maintaining reliable communication and meeting both communication and sensing requirements.

### 5.3. Sensing-Performance Evaluation

In the following sensing simulations, the communication–sensing-power-allocation factor is not explicitly introduced, so that the performance gains provided by the enlarged effective-array aperture can be evaluated independently. The influence of the power-allocation factor on sensing performance is further investigated in the subsequent discussion. For a fair comparison, Physical-4, Virtual-7, and Virtual-10 employ the same transmit waveform, transmit power, target conditions, noise level, and corresponding signal-processing settings within each simulation scenario, with only the array configuration used for angular processing being varied.

The sensing-performance evaluation consists of two scenarios. Scenario I is mainly used to independently evaluate the influence of different array configurations on sensing performance. For this purpose, a two-dimensional coplanar sensing model is adopted, in which the three-dimensional position of the UAV platform is not included in the geometric modeling, and only the relative positions between the array and the three fixed targets within the same sensing plane are considered. The target-detection and angle-domain sensing performance of different array configurations is compared through range–Doppler maps, angle–Doppler maps, and angular spectra.

Scenario II further provides a quantitative evaluation of the angle-domain performance of different array configurations. A simplified static two-target model is adopted, in which the target range and velocity dimensions are not considered and only the spatial angular dimension is retained. By varying the angular separation between the two targets, the DOA-estimation accuracy and closely spaced target-resolution capability of different array configurations are compared.

#### 5.3.1. Detection with Physical and Virtual Arrays

Scenario I considers a fixed three-target scenario to compare the target detection and angle-estimation performance of different array configurations. The aforementioned range–Doppler processing procedure is first applied to obtain the target responses in the range–Doppler domain, followed by conventional beamforming (CBF)-based angle scanning to obtain the corresponding angle–Doppler responses and angular spectra. For a fair comparison, a two-dimensional coplanar geometry is adopted and the vertical height difference is omitted. The three targets have one-way ranges of approximately 65m, 75m, and 95m, with azimuth angles of $-23^{\circ }$, $15^{\circ }$, and $50^{\circ }$, and Doppler frequencies of approximately −250Hz, −80Hz, and 220Hz, respectively. The range–Doppler maps, angle–Doppler maps, and angular spectra of Physical-4, Virtual-7, and Virtual-10 are compared to evaluate the target-detection and angular-resolution performance of the virtual arrays.

(1) Range–Doppler Detection and Peak Selection

**Figure 5:**
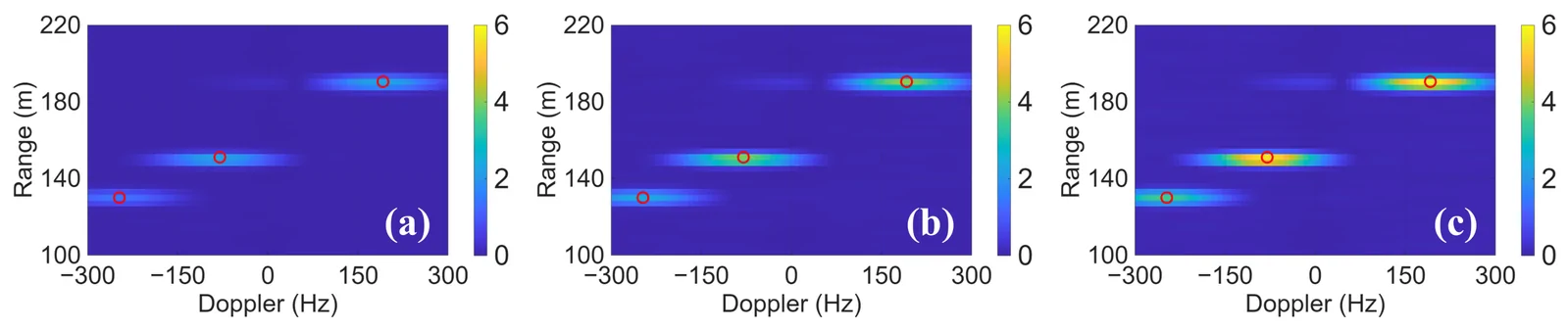
Range–Doppler maps obtained with different array configurations: (**a**) physical 4×1 array, (**b**) virtual 7×1 array, and (**c**) virtual 10×1 array. The red circles denote the detected target peaks. The range axis represents the two-way propagation range.

Figure 5 compares the range–Doppler maps obtained with the physical 4×1 array, virtual 7×1 array, and virtual 10×1 array. A peak-search-based detection method is applied on the RD plane, and the detected target peaks are marked by red circles. The results show that all three array configurations can detect the targets near the predefined range and Doppler positions. Compared with the physical array, the two virtual arrays provide more concentrated energy distributions and clearer peak responses on the RD plane. The target peaks become more prominent for both Virtual-7 and Virtual-10, indicating improved target detectability. Overall, the virtual-array construction enhances energy focusing and peak distinguishability without degrading range–Doppler localization, providing more reliable inputs for subsequent angle estimation and multi-target discrimination.

(2) Angle–Doppler Map Comparison

**Figure 6:**
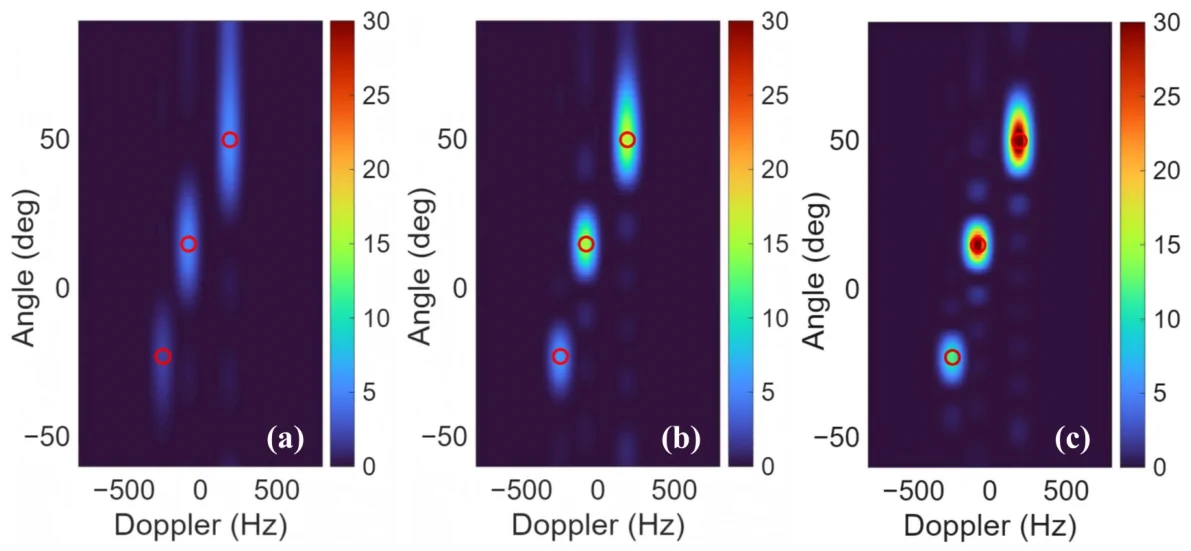
Angle–Doppler maps obtained with different array configurations: (**a**) physical 4×1 array, (**b**) virtual 7×1 array, and (**c**) virtual 10×1 array. The red circles denote the detected target peaks.

Based on the RD peak-detection results, Figure 6 compares the angle–Doppler maps of the physical 4×1, virtual 7×1, and virtual 10×1 arrays, where the red circles indicate the detected target peaks. The three targets are located at approximately $-23^{\circ }$, $15^{\circ }$, and $50^{\circ }$, with Doppler frequencies of about −250Hz, −80Hz, and 220Hz, respectively.

All three arrays successfully detect the targets. Compared with the physical array, the virtual arrays produce more concentrated responses and smaller angular spreading, with the virtual 10×1 array providing the most compact peaks due to its larger effective aperture. These results confirm the improved spatial resolution and multi-target discrimination enabled by virtual-aperture extension.

(3) Angular Focusing and Power Gain

**Figure 7:**
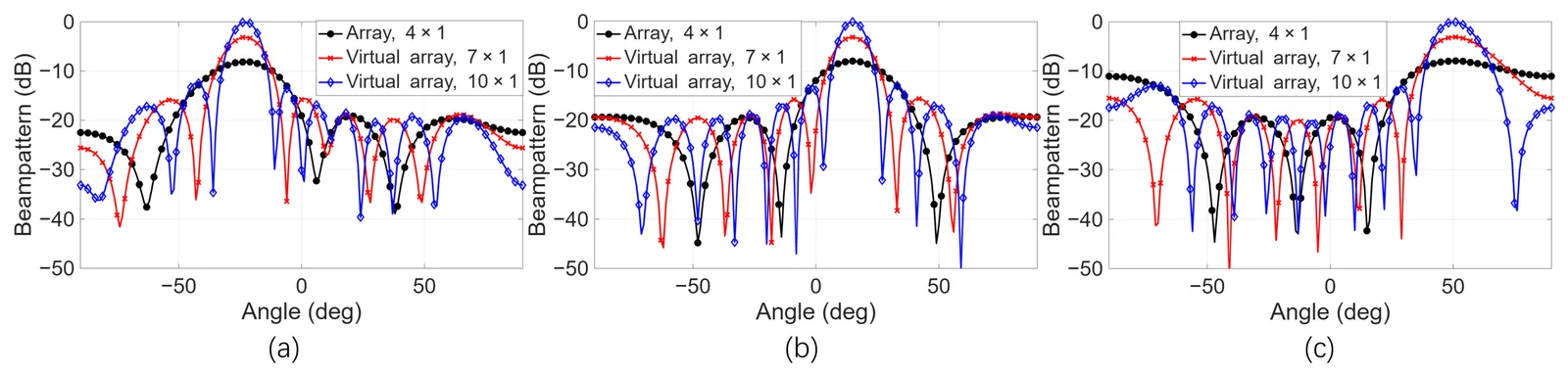
Beampattern comparison of the physical 4×1 array, the virtual 7×1 array, and the virtual 10×1 array under three different target angles. Subfigures (**a**–**c**) correspond to different target angles, illustrating the beamforming characteristics of different array configurations.

The preceding RD-map results show that virtual-array processing enhances target energy concentration while preserving range–Doppler localization. To further evaluate angle-domain performance, Figure 7 compares the angular responses of the physical 4×1, virtual 7×1, and virtual 10×1 arrays, with all curves normalized by the peak gain of the virtual 10×1 array.

All three arrays produce peaks near the true target angles. Compared with the physical array, the virtual arrays exhibit narrower mainlobes and higher peak responses, indicating improved angular resolution and focusing capability. The virtual 10×1 array performs best due to its larger effective aperture, confirming the benefit of virtual-aperture extension for angle-domain sensing.

#### 5.3.2. Statistical-Performance Evaluation

The preceding results show that the performance gains of the virtual arrays are mainly reflected in the angle domain, particularly in terms of energy concentration and angular resolution. Therefore, Scenario II adopts a simplified static two-target model and varies the angular separation between the targets to further evaluate the DOA-estimation accuracy and target-resolution capability of different array configurations. Scenario II is developed on the basis of Scenario I by retaining the common system and array settings while simplifying the sensing model to the spatial angular dimension. In this scenario, only DOA estimation is considered, without evaluating target separability in the range and Doppler dimensions. The MUSIC is employed for high-resolution angle estimation, with the effective complex coefficients of the two targets modeled as statistically uncorrelated across spatial snapshots. Except for the target settings and angle-domain processing, the common system parameters are kept consistent with those in Scenario I, and the number of spatial snapshots used for covariance estimation is set to $L_{s}=64$.

In this scenario, two targets are located at azimuth angles $\vartheta_{1}=30^{\circ }$ and $\vartheta_{2}=20^{\circ }$, corresponding to an angular separation of $\Delta \vartheta =10^{\circ }$. Under different SNR conditions, the physical 4×1 array, virtual 7×1 array, and virtual 10×1 array are compared in terms of DOA RMSE, root CRB, probability of resolution, and conditional DOA RMSE.

**Figure 8:**
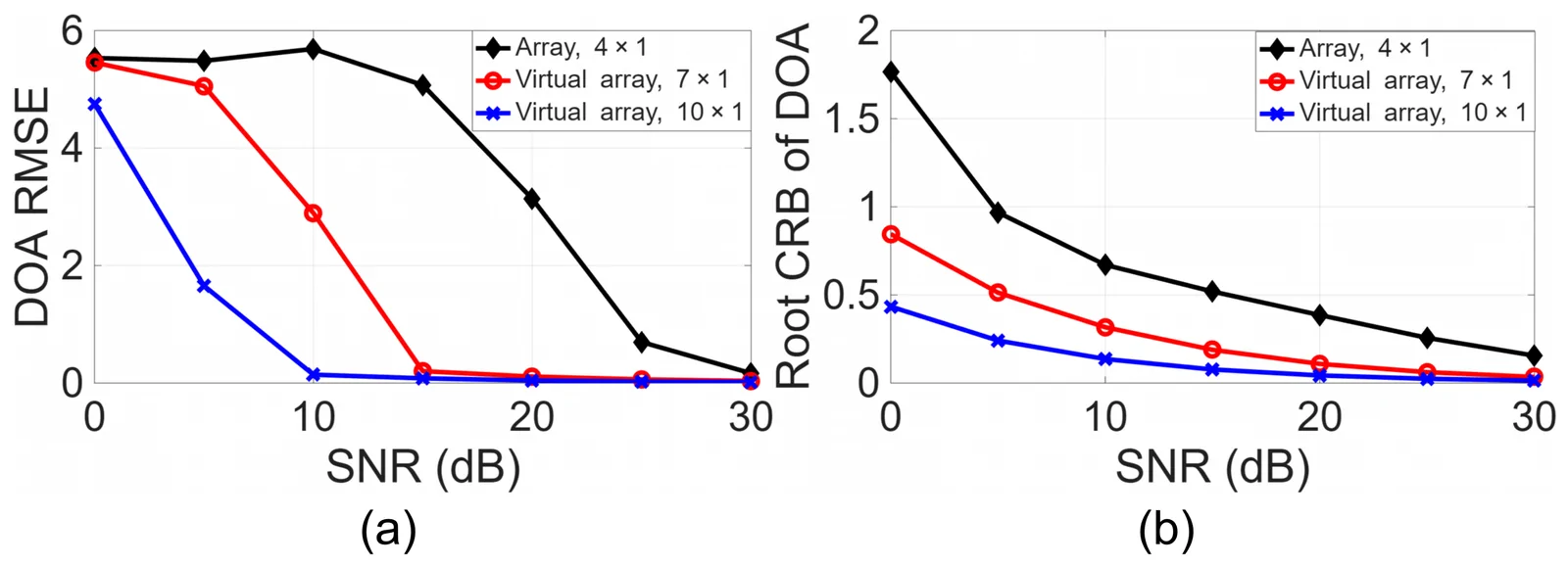
DOA-estimation performance versus SNR in the fixed two-target scenario: (**a**) DOA RMSE and (**b**) root CRB of DOA. The results compare the physical 4×1 array, virtual 7×1 array, and virtual 10×1 array.

**Figure 9:**
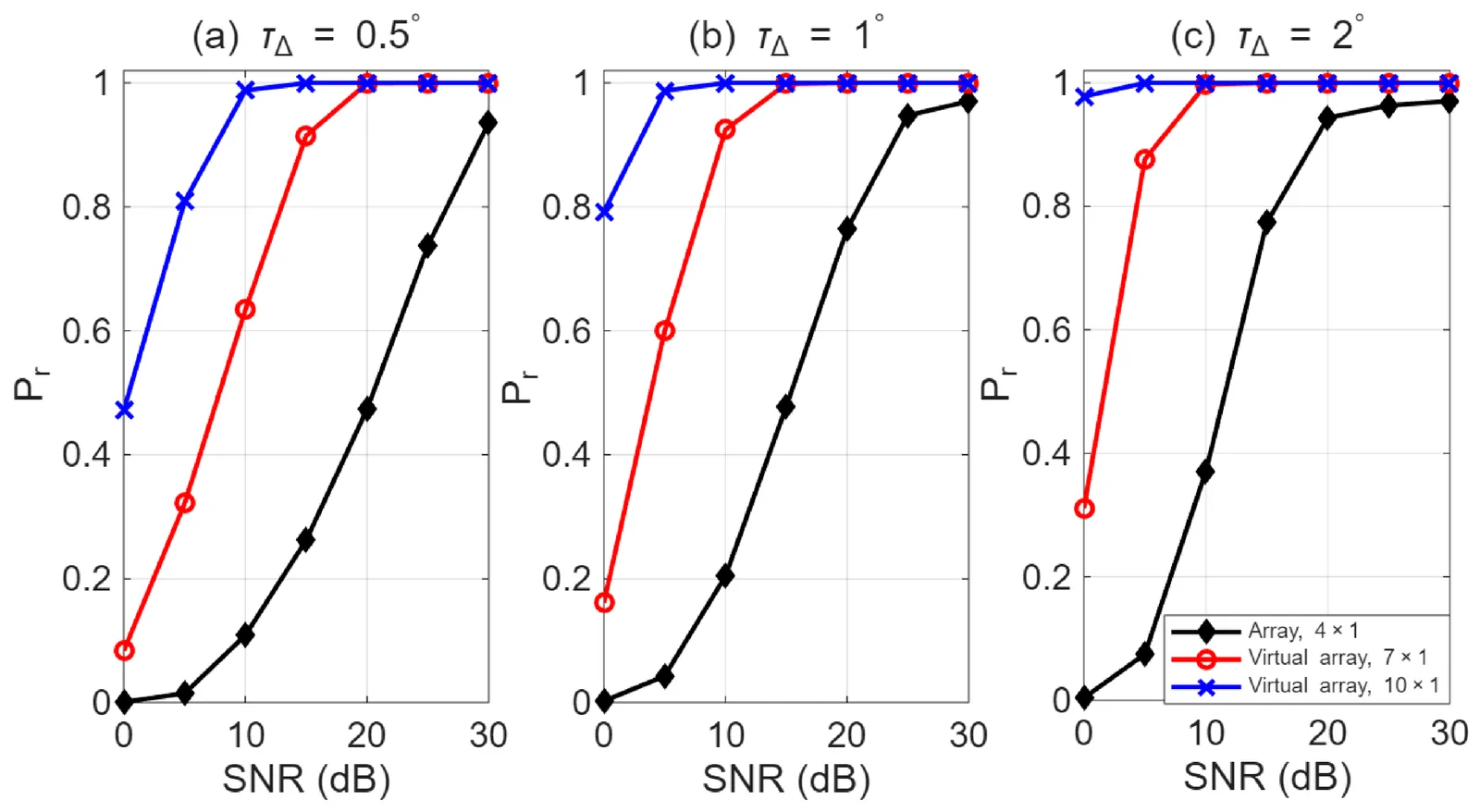
Probability of resolution $P_{r}$ versus SNR under different angular-separation thresholds $\tau_{\Delta }$: (**a**) $\tau_{\Delta }=0.5^{\circ }$, (**b**) $\tau_{\Delta }=1^{\circ }$, and (**c**) $\tau_{\Delta }=2^{\circ }$. The results compare the physical 4×1 array, virtual 7×1 array, and virtual 10×1 array.

**Figure 10:**
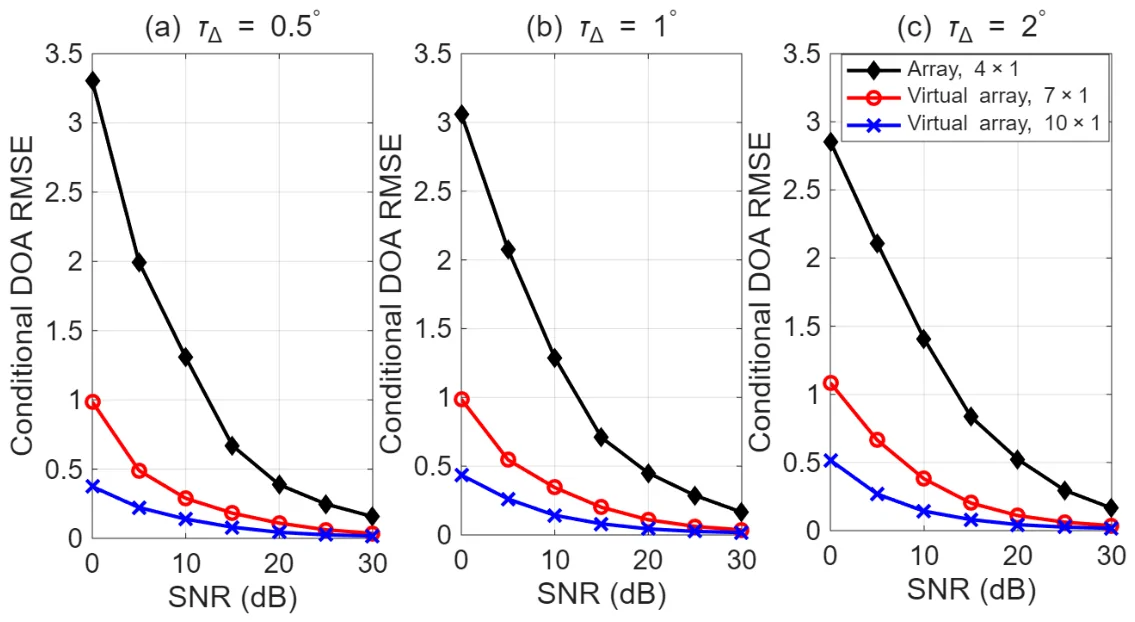
Conditional DOA RMSE versus SNR under different angular-separation thresholds $\tau_{\Delta }$: (**a**) $\tau_{\Delta }=0.5^{\circ }$, (**b**) $\tau_{\Delta }=1^{\circ }$, and (**c**) $\tau_{\Delta }=2^{\circ }$. The results compare the physical 4×1 array, virtual 7×1 array, and virtual 10×1 array.

Figure 8, Figure 9 and Figure 10 show the DOA-estimation performance of different array configurations in the fixed two-target scenario. As the SNR increases, the DOA-estimation performance of all array configurations generally improves, while the virtual arrays consistently outperform the physical array.

Specifically, as shown by the DOA RMSE and root CRB results, for the Physical-4, Virtual-7, and Virtual-10 configurations, the DOA RMSE remains above the corresponding root CRB over the entire simulated SNR range, including the high-SNR region. As the SNR increases, the RMSE of all three configurations gradually approaches the corresponding theoretical lower bound but does not fall below it. Among the three configurations, Virtual-10 achieves the lowest DOA RMSE and root CRB, followed by Virtual-7, whereas Physical-4 exhibits the largest estimation error.

The probability-of-resolution results further show that the two-target resolution capability of all three arrays improves significantly as the SNR increases. For the considered angular-separation thresholds $\tau_{\Delta }$, Virtual-10 consistently achieves the highest $P_{r}$, followed by Virtual-7, whereas Physical-4 exhibits the lowest probability of resolution. In addition, increasing $\tau_{\Delta }$ relaxes the resolution criterion and, therefore, generally leads to a higher $P_{r}$ at the same SNR.

The conditional DOA RMSE results exhibit a consistent performance ordering among the three array configurations. As the SNR increases, the conditional DOA RMSE evaluated over the successfully resolved trials decreases for all three arrays. Virtual-10 consistently achieves the lowest conditional estimation error, followed by Virtual-7, while Physical-4 exhibits the highest error. The consistent trends observed under different $\tau_{\Delta }$ values further indicate that the virtual arrays maintain improved DOA-estimation accuracy and two-target-resolution performance under different resolution criteria.

**Figure 11:**
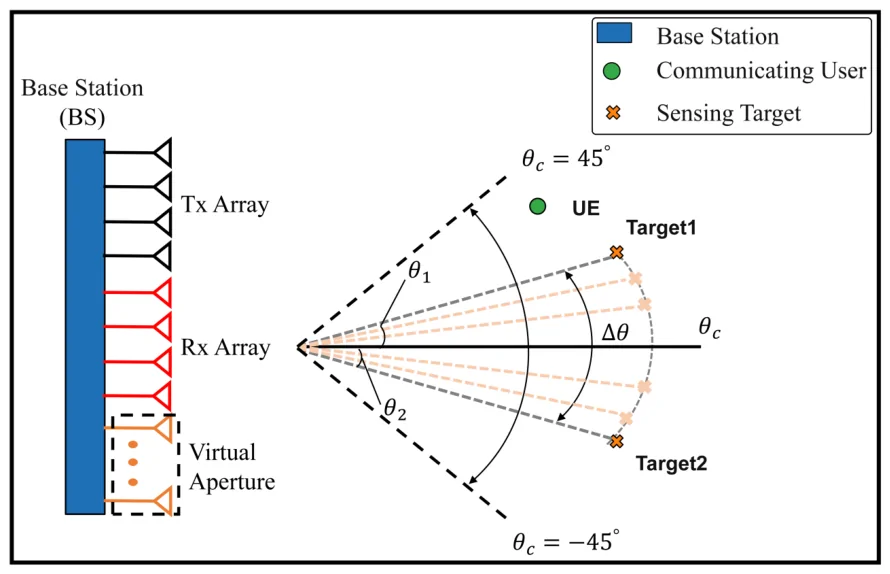
Scenario illustration of the angular-separation variation between two sensing targets. The angular center $\theta_{c}$ is scanned from $-45^{\circ }$ to $45^{\circ }$, while the angular separation Δθ between the two targets varies from $5^{\circ }$ to $25^{\circ }$.

As illustrated in Figure 11, Scenario II considers a general two-target sensing case, where the angular center $\theta_{c}$ and angular separation Δθ are jointly varied. To avoid dependence on a specific pair of target directions, a statistical evaluation is performed by jointly scanning $\theta_{c}$ and Δθ with Monte Carlo simulations.

The angular center and angular separation are selected as $\theta_{c}\in \left\{-45^{\circ },-40^{\circ },\ldots ,45^{\circ }\right\}$ and $\Delta \theta \in \{5^{\circ },6^{\circ },\ldots ,25^{\circ }\}$, respectively. For each pair of $\theta_{c}$ and Δθ, the two target angles are defined as $\theta_{1}=\theta_{c}-\Delta \theta /2$ and $\theta_{2}=\theta_{c}+\Delta \theta /2$. This gives 19×21=399 cases. For each case, $N_{\mathrm{MC}}=2000$ Monte Carlo trials are performed. For each angular separation, the simulation results obtained from all valid angular centers are pooled to compute the overall statistical-performance metrics.

**Figure 12:**
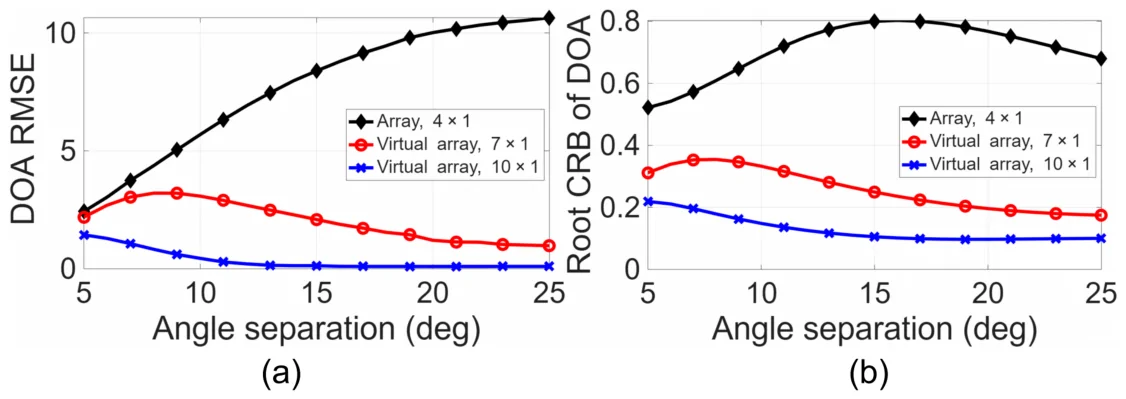
DOA-estimation performance versus angular separation at SNR=10 dB: (**a**) DOA RMSE and (**b**) root CRB of DOA. The results are averaged over the considered angular-center directions and compare the physical 4×1 array, virtual 7×1 array, and virtual 10×1 array.

**Figure 13:**
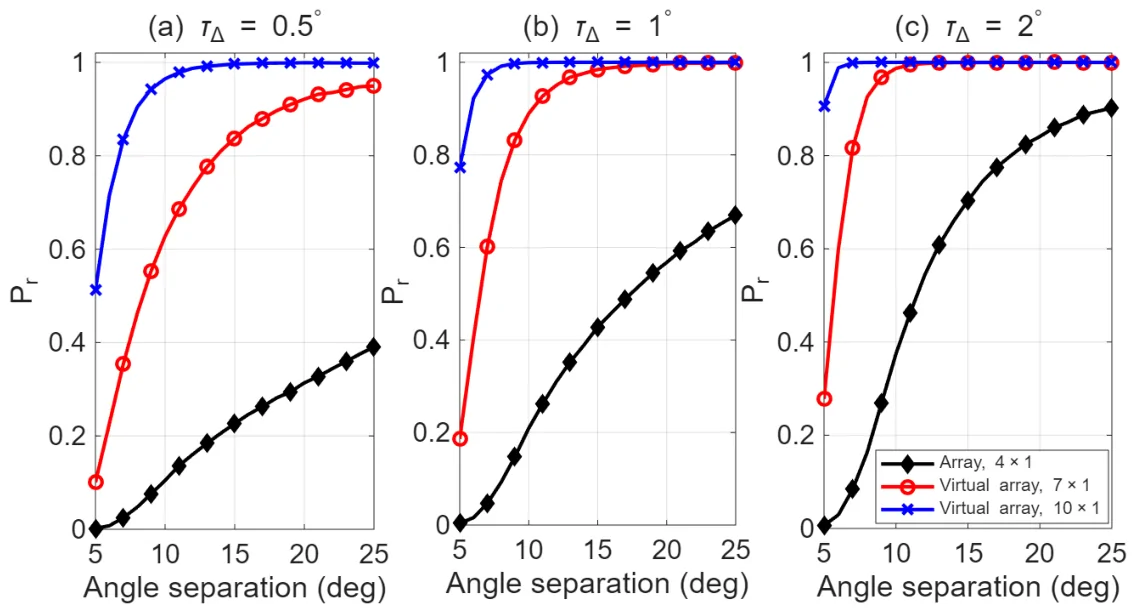
Probability of resolution $P_{r}$ versus angular separation at SNR=10 dB under different angular-separation thresholds $\tau_{\Delta }$: (**a**) $\tau_{\Delta }=0.5^{\circ }$, (**b**) $\tau_{\Delta }=1^{\circ }$, and (**c**) $\tau_{\Delta }=2^{\circ }$. The results compare the physical 4×1 array, virtual 7×1 array, and virtual 10×1 array.

**Figure 14:**
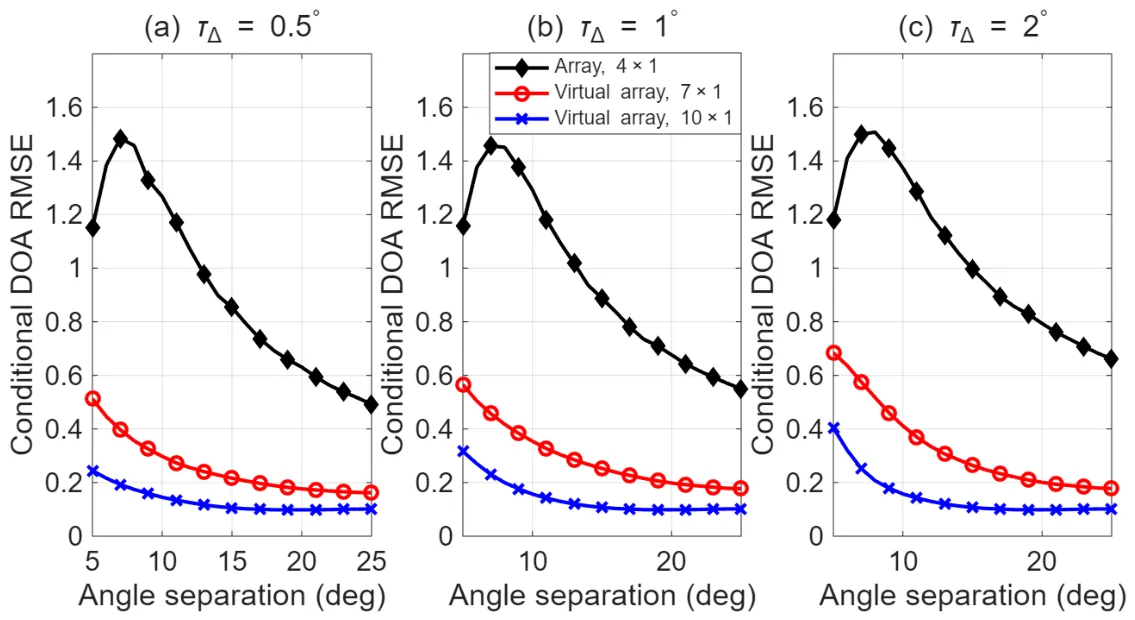
Conditional DOA RMSE versus angular separation at SNR=10 dB under different angular-separation thresholds $\tau_{\Delta }$: (**a**) $\tau_{\Delta }=0.5^{\circ }$, (**b**) $\tau_{\Delta }=1^{\circ }$, and (**c**) $\tau_{\Delta }=2^{\circ }$. The results compare the physical 4×1 array, virtual 7×1 array, and virtual 10×1 array.

Figure 12, Figure 13 and Figure 14 present the statistical DOA-estimation performance versus angular separation at SNR=10 dB.

As shown in Figure 12a,b, the two virtual arrays generally achieve lower DOA RMSE and root CRB than the physical array over the entire considered angular-separation range, with Virtual-10 performing best, followed by Virtual-7. It should be noted that the conventional DOA RMSE in Figure 12a does not decrease monotonically with angular separation. This is because the conventional RMSE is calculated over all Monte Carlo trials, and its variation depends not only on whether the two targets are successfully resolved but also on the magnitude of the estimation errors in unsuccessful or incorrectly matched trials. When the two targets are not correctly resolved or are incorrectly matched, a larger true angular separation may result in a larger numerical angular error. Consequently, the DOA RMSE of Physical-4 may increase over part of the angular-separation range.

As shown in Figure 12b, Virtual-10 generally provides the lowest theoretical error bound, followed by Virtual-7, whereas Physical-4 exhibits the highest root CRB. The root-CRB curves do not necessarily follow a strictly monotonic trend because the result at each angular separation is obtained by pooling over different angular-center configurations. As the angular separation varies, the actual target directions, target-parameter coupling, and angular sensitivity of the array also change. Therefore, the root CRB averaged over different angular-center configurations does not necessarily vary monotonically with angular separation.

Figure 13 further presents the probability of resolution $P_{r}$ under different angular-separation thresholds $\tau_{\Delta }$. As the true angular separation increases, $P_{r}$ generally increases for all three array configurations, indicating that a larger angular separation improves the two-target resolution capability. For all considered values of $\tau_{\Delta }$, Virtual-10 consistently achieves the highest probability of resolution and approaches unity at a relatively small angular separation. Virtual-7 provides the second-best performance, whereas Physical-4 requires a larger angular separation to achieve a high probability of resolution. In addition, increasing $\tau_{\Delta }$ relaxes the successful-resolution criterion and, therefore, generally leads to a higher $P_{r}$ at the same true angular separation.

Figure 14 shows the conditional DOA RMSE calculated only over the successfully resolved trials. For Virtual-7 and Virtual-10, the conditional DOA RMSE generally decreases as the angular separation increases, with Virtual-10 maintaining the lowest conditional estimation error under the different $\tau_{\Delta }$ values, followed by Virtual-7. The conditional DOA RMSE of Physical-4 exhibits some non-monotonic variation in the small-angular-separation region and then generally decreases as the angular separation increases. This behavior arises because the conditional DOA RMSE is evaluated only over trials satisfying the successful-resolution criterion. As the angular separation changes, the set of trials included in the conditional statistics also changes, and, therefore, the conditional RMSE does not necessarily exhibit a strictly monotonic trend. Nevertheless, the two virtual arrays generally maintain lower conditional estimation errors than Physical-4 over the considered angular-separation range.

Overall, Virtual-10 achieves the best performance in terms of conventional DOA RMSE, root CRB, probability of resolution $P_{r}$, and conditional DOA RMSE, followed by Virtual-7. These results demonstrate that the effective aperture advantage provided by the virtual arrays can improve two-target DOA-estimation accuracy, reduce the theoretical error bound, and enhance the resolution capability for closely spaced targets.

## 6. Discussion

### 6.1. UAV-Aided Orthogonal Virtual Array

**Figure 15:**
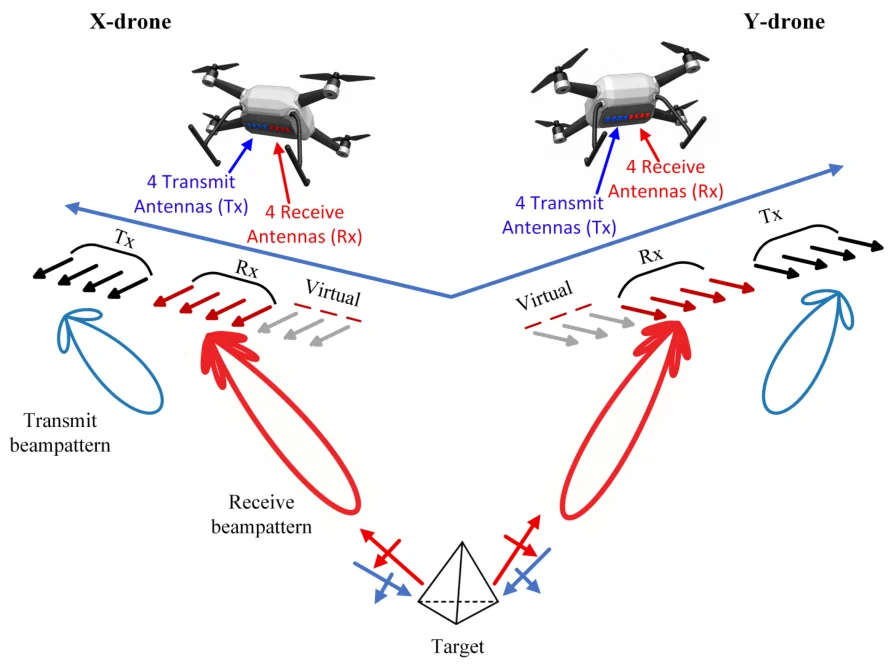
Orthogonal configuration of the X-drone and Y-drone dual-polarized antenna arrays. The array orientations carried by the two UAVs are mutually orthogonal to provide complementary spatial observations for target sensing.

As shown in Figure 15, the proposed system adopts an orthogonal dual-UAV configuration composed of the X-drone and Y-drone. By exploiting the position and attitude adjustability of the UAVs in three-dimensional space, a local coplanar orthogonal sensing configuration can be established for the considered target geometry, where the array orientations carried by the two UAVs are mutually orthogonal.

Each UAV is equipped with four transmit antennas and four receive antennas. By combining the Tx/Rx antennas, virtual arrays can be formed along two orthogonal array directions, thereby extending the equivalent sensing aperture without significantly increasing the antenna number on a single UAV platform.

**Figure 16:**
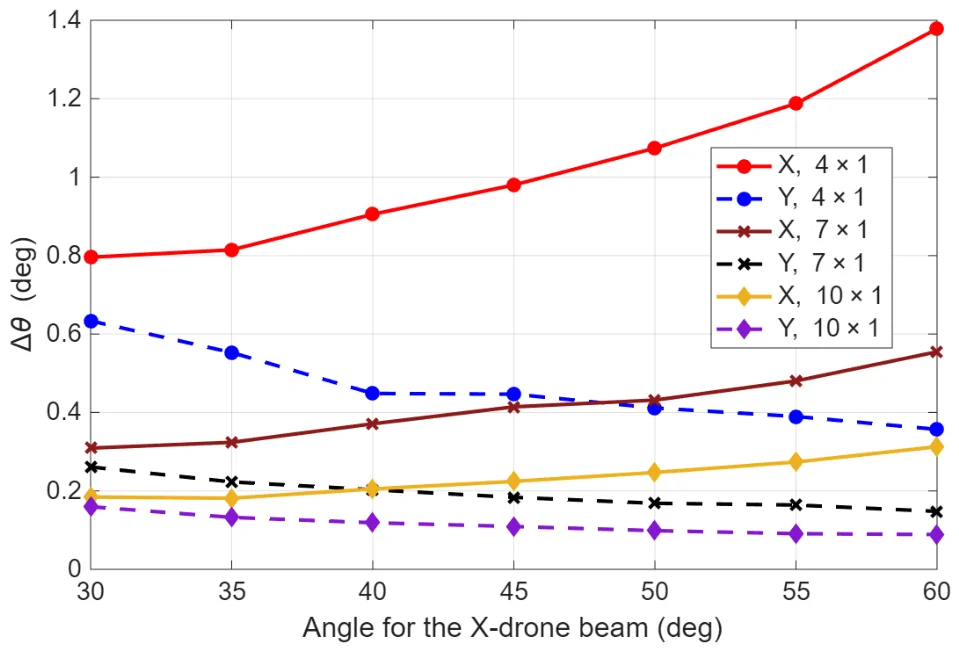
Mean absolute angle-estimation error versus the X-drone beam angle $\theta_{x}$ under different array configurations. For the same target direction, the Y-drone has a different local beam angle due to the orthogonal observation geometry, where 4×1 denotes the physical array and 7×1 and 10×1 denote virtual-array configurations.

Figure 16 presents the mean absolute angle-estimation error of the X-drone and Y-drone in a single-target scenario under the coplanar orthogonal configuration described above, where each point is averaged over 1000 Monte Carlo trials. Compared with the 4×1 physical array, the 7×1 and 10×1 virtual arrays achieve lower estimation errors over most of the considered angular range, and the 10×1 virtual array provides the best overall performance. This indicates that virtual-array extension can enlarge the effective sensing aperture and improve the DOA-estimation accuracy.

In the considered coplanar orthogonal configuration, the positive global *x*-axis and *y*-axis are adopted as the local angular reference directions for the X-drone and Y-drone, respectively. Accordingly, $\theta_{x}$ and $\theta_{y}$ denote the local target angles measured with respect to these two orthogonal reference directions. Therefore, the different error trends of the X-drone and Y-drone reflect the complementary angular observations provided by the orthogonal dual-UAV configuration, which helps alleviate the limitation of single-direction sensing and improve the reliability of target sensing and localization.

### 6.2. Impact of the Trade-Off Coefficient on Sensing

To further investigate the impact of the communication–sensing power allocation factor α on two-target sensing performance, the probability of resolution, denoted by $P_{r}$, is evaluated under several representative two-target scenarios. The angular-separation error tolerance is set to $\tau_{\Delta }=1^{\circ }$ in this evaluation. Here, α denotes the fraction of transmit power allocated to communication, while the remaining fraction 1−α is allocated to sensing. Four typical angular separations, namely $5^{\circ }$, $10^{\circ }$, $15^{\circ }$, and $20^{\circ }$, are considered, together with five azimuth-center directions of $-40^{\circ }$, $-20^{\circ }$, $0^{\circ }$, $20^{\circ }$, and $40^{\circ }$. For each parameter combination, the two targets are symmetrically located on both sides of the azimuth center, and the resulting resolution probabilities are averaged over all considered configurations to reduce the influence of a specific target location.

**Figure 17:**
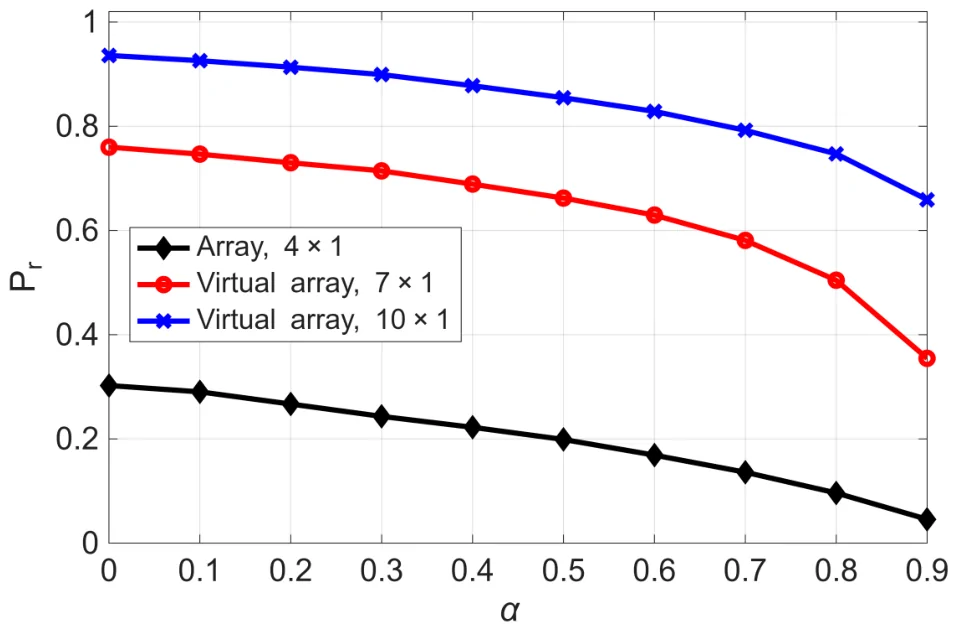
Probability of successful two-target resolution $P_{r}$ versus the communication–sensing-power-allocation factor α for the physical 4×1 array and the virtual 7×1 and 10×1 arrays under $\tau_{\Delta }=1^{\circ }$. The results are averaged over typical angular separations and azimuth-center directions.

As shown in Figure 17, $P_{r}$ generally decreases as α increases. This is because a larger communication-power fraction reduces the effective sensing SNR, making the two MUSIC peaks more likely to merge, be missed, or deviate from the true target directions. Among the three array configurations, the virtual 10×1 array consistently achieves the highest $P_{r}$, followed by the virtual 7×1 array and the physical 4×1 array.

These results indicate that virtual-array processing improves two-target angular resolution by enlarging the effective aperture and can partially mitigate the performance loss caused by reduced sensing power. Nevertheless, the resolution probability still decreases at large α, indicating that the power-allocation factor should be properly selected to balance communication performance and two-target sensing reliability.

## 7. Conclusions

In this paper, a virtual-array-implementation method was proposed for UAV-aided MIMO-OFDM ISAC systems to expand the effective aperture and improve angle-domain sensing capability under a compact configuration requirement for hardware. Simulation results show that, compared with the physical 4×1 array, the virtual 7×1 and 10×1 arrays improve DOA-estimation accuracy, lower the error bound, and enhance detection resolution in terms of distinguishing multiple targets, with the virtual 10×1 array achieving the best performance. The orthogonal dual-UAV configuration further provides spatial diversity and improves sensing robustness. It provides an avenue for UAV-ISAC systems to be deployed in search and rescue applications where multiple targets need to be detected with a high angular accuracy. 

## Figures and Tables

**Table 1:** Main System Simulation Parameters.

Parameter	Value	Description
$f_{c}$	6GHz	Carrier frequency
λ	0.05m	Carrier wavelength
*B*	100MHz	Signal bandwidth
$f_{s}$	100.004MHz	Nominal complex-baseband sampling rate
$N_{c}$	2048	Number of OFDM subcarriers
Δf	48.83kHz	Subcarrier spacing
$N_{\mathrm{CP}}$	200	Cyclic-prefix length
$T_{\mathrm{CP}}$	2.00μs	Cyclic-prefix duration
$N_{t}$	4	Number of transmit antennas
$N_{r}$	4	Number of receive antennas
NF	3dB	Receiver noise figure
$L_{o}$	4	Length of orthogonal sensing-pilot sequence
*M*	64	QAM modulation order
$N_{s}$	20	Number of OFDM symbols
$N_{\mathrm{MC}}$	2000	Number of Monte Carlo trials
*c*	$3\times 10^{8}\;m/s$	Speed of light

**Table 2:** Physical-Array-Geometry Parameters.

Configuration	$d_{\mathrm{tx}}$	$d_{\mathrm{rx}}$	$d_{s}$
Physical-4	25mm	25mm	50mm
Virtual-7	25mm	25mm	50mm
Virtual-10	25mm	50mm	50mm

## Data Availability

All data generated or analyzed during this study are included in this published article.
